# Patient‐Derived IgG Epitope Mapping of Bet v 1 Reveals Hypoallergenic Peptide Candidates for Safe and Next‐Generation Allergen Immunotherapy

**DOI:** 10.1111/cea.70276

**Published:** 2026-03-10

**Authors:** Lara Šošić, Marta Paolucci, Alessandro Streuli, David A. Melillo, Tianchi Jiang, Raffaela Campana, Claudia C. V. Lang, Thomas M. Kündig, Christian Steuer, Klaus Eyer, Pål Johansen

**Affiliations:** ^1^ Department of Dermatology University of Zurich Schlieren Switzerland; ^2^ Laboratory for Functional Immune Repertoire Analysis, Institute of Pharmaceutical Sciences, Department of Chemistry and Applied Biosciences ETH Zurich Zurich Switzerland; ^3^ Laboratory for Pharmaceutical Analytics, Institute of Pharmaceutical Sciences, Department of Chemistry and Applied Biosciences ETH Zurich Zurich Switzerland; ^4^ Division of Immunopathology, Department of Pathophysiology and Allergy Research, Center for Pathophysiology, Infectiology and Immunology Medical University of Vienna Vienna Austria; ^5^ Department of Dermatology University Hospital Zurich Zurich Switzerland; ^6^ Department of Biomedicine Aarhus University Aarhus Denmark

**Keywords:** allergen immunotherapy, antibodies, basophil activation assay, DropMap, epitope mapping, synthetic peptides

## Abstract

**Background:**

Allergen immunotherapy (AIT) is the only curative treatment for allergic diseases, primarily by inducing allergen‐neutralising IgG antibodies. However, its use is limited by frequent allergic adverse events.

**Objective:**

To facilitate the design of IgG‐epitope‐based peptide vaccines, this study aimed to identify IgG‐binding regions on the major birch pollen allergen, *Bet v 1*, using patient‐derived immune profiles.

**Methods:**

Blood samples were collected from 30 birch pollen‐allergic patients, both AIT‐treated and untreated; healthy individuals were included as controls. Sera were analysed for allergen‐specific IgE, IgG, and IgG4, and basophil degranulation inhibition was assessed in humanised RBL cells and blood from allergic individuals. We used DropMap microfluidics for single‐cell affinity profiling of IgG‐ and IgG4‐secreting B cells. IgG epitopes were mapped using overlapping peptides and CLIPS technology. Candidate AIT peptides were synthesised on solid phase via Fmoc strategy, and their allergenicity was assessed in basophil degranulation and cellular antigen stimulation assays.

**Results:**

Sera from AIT‐treated patients exhibited elevated IgG and IgG4 levels and enhanced inhibition of basophil degranulation. Single‐cell analysis indicated IgG‐affinity maturation with AIT. IgG‐epitope mapping identified four distinct, non‐overlapping IgG‐binding regions on *Bet v 1*. Six peptides derived from these regions were successfully produced and did not induce basophil degranulation in vitro. The application of DropMap microfluidics allowed high‐resolution single B‐cell analysis, providing novel insights into allergen‐specific B‐cell responses and the effects of AIT at the clonal level.

**Conclusion:**

This integrated approach identifies hypoallergenic peptides that selectively engage protective IgG responses and offers a framework for developing safer, next‐generation immunotherapies for allergic disease.

## Key Messages

- Epitope mapping identified IgG‐binding regions on *Bet‐v‐1*, providing basis for a rational AIT design.
- Single‐cell DropMap analysis revealed AIT‐associated affinity maturation of allergen‐specific IgG‐secreting B cells.
- Peptides from IgG‐binding regions showed low allergenicity, supporting further evaluation as AIT candidate components.

## Graphical abstract

Mapping IgG epitopes of the major birch allergen Bet v 1 identified patient‐derived, hypoallergenic peptides that did not trigger degranulation. These findings support a novel, safer approach for peptide‐based allergen immunotherapy that leverages naturally induced IgG specificities from allergic individuals.

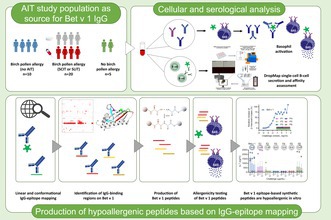

## 1 Introduction

Allergen immunotherapy (AIT) is the only disease‐modifying treatment for allergic diseases, aiming to induce long‐term immune tolerance through repeated allergen exposure [1, 2]. A key mechanism involves the induction of allergen‐specific IgG antibodies that compete with IgE for allergen binding, thereby preventing mast cell and basophil activation [3].

Recent advances in passive immunotherapy using allergen‐specific monoclonal IgG4 antibodies have demonstrated that pre‐emptively blocking major allergens can suppress allergic responses in humans with remarkable efficacy [4, 5, 6, 7]. These results highlight the therapeutic potential of allergen‐neutralising IgG antibodies and motivate the rational design of vaccines that favour IgG responses over IgE activation [8].

However, current AIT formulations often rely on native or minimally modified allergens, which carry a high risk of adverse IgE‐mediated reactions. To enhance safety, several hypoallergenic strategies have been explored [9], including chemically modified allergens [10, 11], recombinant variants [12], and synthetic peptides [13, 14]. Despite these efforts, most designs do not specifically leverage patient‐derived IgG binding profiles, nor do they systematically exclude IgE‐reactive regions. Moreover, the precise epitope landscape of protective IgG antibodies induced during AIT remains incompletely defined, and the clonal and functional relationship between IgE‐ and IgG‐producing B cells is poorly understood [15].

While AIT has been proven effective for many IgE‐mediated allergies [1, 2], a substantial proportion of allergic patients either fail to initiate or do not complete AIT [16, 17]. A broader application of AIT is hindered by the risk of allergic side effects, including systemic anaphylaxis, as well as by inconsistent treatment responses. The development of safer and more effective AIT formulations—particularly those that minimize IgE reactivity while promoting allergen‐neutralising IgG—could help overcome this therapeutic gap.

In this study, we developed an integrative, patient‐derived approach to identify and characterise IgG‐binding regions of the major birch pollen allergen *Bet v 1*. Using a combination of high‐resolution epitope mapping (including constrained peptide scaffolds), single‐cell DropMap microfluidics, and functional in vitro allergenicity assays, we profiled antibody repertoires in AIT‐treated and untreated allergic individuals. Based on these data, we designed and synthesised hypoallergenic peptides that should selectively engage protective IgG responses without inducing IgE‐mediated side effects through basophil activation. Our findings lay the groundwork for a new class of precision‐designed, peptide‐based allergen immunotherapies with improved safety and mechanistic insight.

## 2 Materials and Methods

### 2.1 Clinical Trial Participants and Human Ethics

**FIGURE 1:**
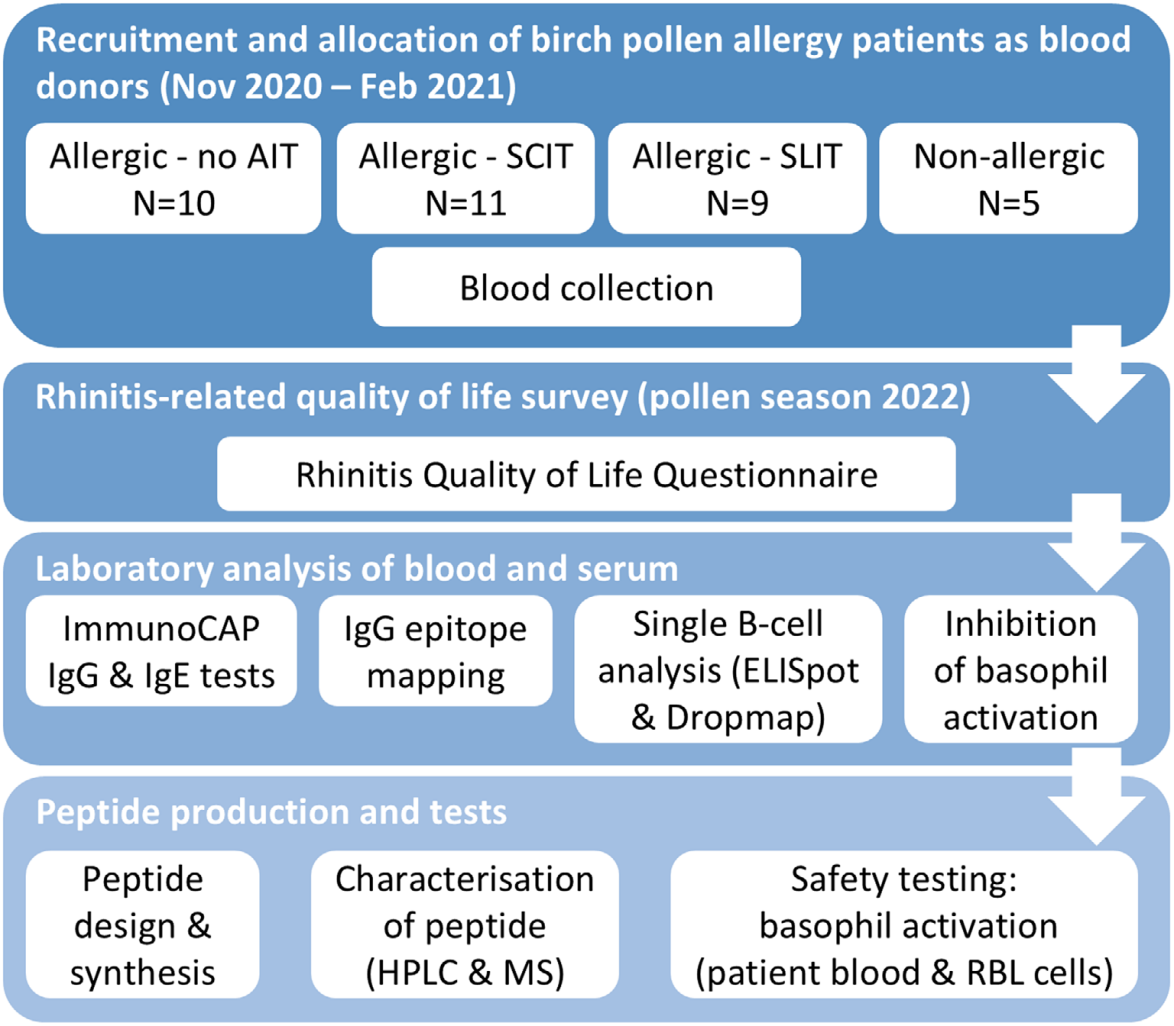
Study outline. Venous blood collection from a total of 30 study participants allergic to birch pollen undergoing SCIT, SLIT or no AIT. Five control participants were not sensitised to birch pollen allergens. Analyses performed were RQLQ assessment of seasonal symptoms, ImmunoCAP of total IgE and *Bet v 1*‐specific IgE, IgG and IgG4, IgG epitope mapping of patient serum on *Bet v 1*, B‐cell analysis, and testing of patient serum blocking capacity with rat basophil leukaemia cell (RBL cell) degranulation assay. Peptides were designed based on IgG epitope mapping and produced for testing in vitro and in mice.

The clinical study included 30 birch pollen‐allergic patients and five non‐allergic (non‐Bet v 1‐sensitised) controls (Figure 1). Blood samples (20 mL EDTA, 20 mL serum) were collected between November 2020 and March 2021. Birch pollen allergy was defined by seasonal allergic rhino‐conjunctivitis symptoms over at least the past two consecutive years, confirmed by serological or cutaneous sensitisation. Exclusion criteria included systemic immunosuppressive or antihistamine treatment within 14 days, chronic inflammatory diseases, current infections, or substance abuse. Participants were recruited from the Allergy Ward, University Hospital Zurich. The study was approved by the Independent Ethics Committee of Kanton Zurich (BASEC‐nos 2020‐01470 and 2023‐01405) and conducted in accordance with the Declaration of Helsinki. All participants provided written informed consent.

All other and below‐listed methods are described in the Supporting Information.

- Rhinitis Quality of Life Questionnaire (RQLQ)
- Blood processing and analysis
- Secretion and antigen specificity analysis of IgE‐secreting B cells in ELISpot.
- DropMap single‐cell analysis of IgG‐secreting memory B cells.
- IgG epitope mapping of *Bet v 1*.
- Production of monoclonal anti‐*Bet v 1* IgE and IgG.
- RBL degranulation and inhibition assays.
- Peptide allergenicity assessment in human blood.
- Expression and purification of recombinant *Bet v 1*.
- Statistical analysis

## 3 Results

### 3.1 Demographical Analysis of Study Cohort

The overall study design and patient flow is shown in Figure 1. A total of 35 participants were enrolled in the study, comprising 20 females (57%) and 15 males (43%) (Table 1, Table S1). The median age was 36 years (range: 18–56). Among the participants, 30 were sensitised and allergic to birch pollen, confirmed by skin‐prick testing, *Bet v 1*‐specific IgE levels, and patient history. Of these allergic individuals, 11 were receiving subcutaneous immunotherapy (SCIT), nine were undergoing sublingual immunotherapy (SLIT), and 10 were not receiving any AIT (“allergic”). None of the participants that received SCIT or SLIT had previously received same or other AIT. Additionally, five non‐Bet v 1‐sensitised, non‐allergic participants were included as controls.

**TABLE 1:** Demographics and baseline characteristics of study participants and serum samples. Mean values (with min‐max range) are shown if not otherwise indicated.

Characteristics	Birch‐allergic patients				Non‐allergic
	No AIT	SCIT^a^	SLIT^b^	±AIT	No AIT
*n*	10	11	9	30	5
Females (%)	6 (60)	5 (45)	6 (67)	17 (57)	3 (60)
Age, median	30 (26–46)	32 (21–43)	35 (18–56)	35 (18–56)	35 (21–41)
AIT cycles^c^	0 (0–0)	2.4 (1–4)	2.3 (2–3)	n.a.	n.a.
tIgE (kU/L)	132 (7–387)	302 (16–853)	176 (16–481)	207 (7–853)	89 (0–320)
CAP class, birch	3.0 (2–5)	3.6 (2–6)	3.7 (1–6)	3.4 (1–6)	0 (0–0)
sIgE (kUa/L)	13 (1–57)	32 (2–159)	49 (1–161)	31	0 (0–0)
sIgG (μg/L)	1.89 (0.88–5.93)	3.58 (0.98–8.62)	3.11 (0.79–5.97)	2.88 (0.79–8.62)	0.7 (0.3–1.1)
sIgG4 (μg/L)	0.21 (0.03–0.52)	1.41 (0.06–2.87)	0.91 (0.00–2.24)	0.86 (0.00–2.87)	0.00 (0.00–0.01)
RQLQ, mean	69 (23–142)^d^	41 (0–80)^e^	54 (7–92)^f^	53 (0–142)^g^	18 (0–28)^h^
RQLQ, median	56	38	62	49	21
RBL inhibition (%)^i^	6 (0–36)	48 (0–99)	54 (0–96)	36 (0–99)	18 (0–28)

^a^ Pre‐seasonal SCIT with Allergovit (Allergopharma), Alutard (ALK‐Abello), or Polvac (Bencard) grass pollen allergen/allergoid extracts.

^b^ Daily SLIT with Grazax (ALK‐Abello) or Oralair (Stallergenes).

^c^ 1 One cycle corresponds to 1 year of completed pre‐seasonal SCIT (6–8 injections) or one whole year of daily SLIT.

^d^ *n* = 8.

^e^ *n* = 10.

^f^ *n* = 7.

^g^ *n* = 25.

^h^ *n* = 4.

^i^ Sensitised RBL cells were incubated with patient sera before Bet v 1 challenge; Increased inhibitions (in %) indicates reduced RBL degranulation.

### 3.2 AIT Enhances Bet v 1‒Specific IgG/IgG4 and Reduces IgE‐Secreting Memory B Cells

**FIGURE 2:**
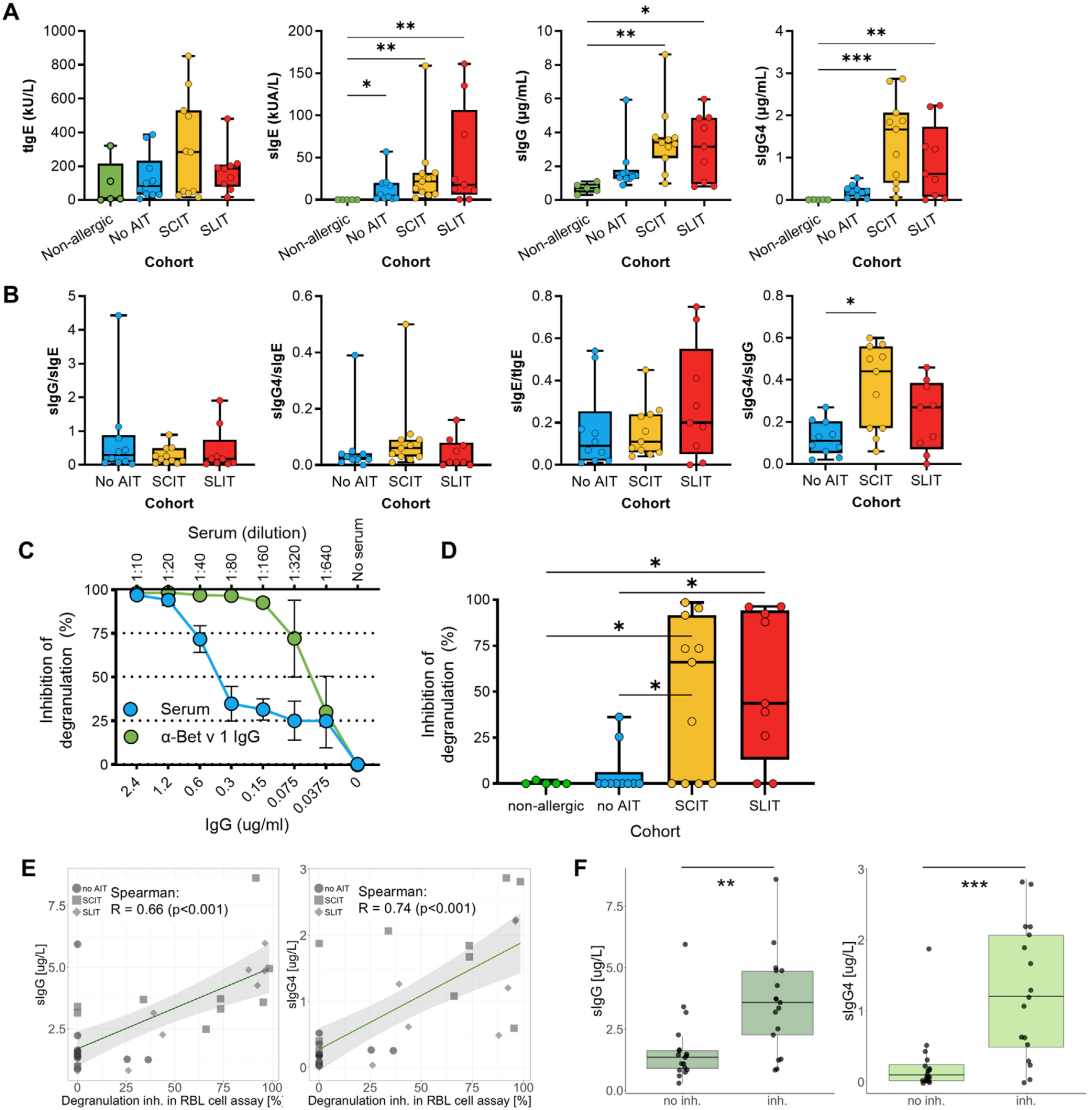
Serological and functional profiling of allergic patients with and without allergen immunotherapy. (A) Concentrations of total IgE (tIgE, kU/L), *Bet v 1*‐specific IgE (sIgE, kUA/L), IgG (sIgG, μg/L), and IgG4 (sIgG4, μg/L) measured in 10 allergic patients without AIT (No AIT), 20 allergic patients with AIT (SCIT, *n* = 11; SLIT, *n* = 9), and five non‐allergic controls (Non‐allergic). (B) Antibody ratios between allergic patients without (*n* = 10) and with AIT (*n* = 20). (C) Inhibition of β‐hexosaminidase release from sensitised RBL cells in the presence of recombinant *Bet v 1*‐specific IgG or serum samples from allergic patients that received SLIT. (D) Percentage of β‐hexosaminidase release inhibition with serum from allergic patients without AIT (No AIT), allergic patients with SCIT or SLIT (AIT), and non‐allergic individuals (Non‐allergic). All sera were tested at a 1:10 dilution. (E) Spearman correlation of sIgG or sIgG4 with β‐hexosaminidase inhibition across all allergic patients. (F) sIgG and sIgG4 concentrations in the serum of all allergic patients, grouped by their ability to inhibit β‐hexosaminidase release (inh.) or not (no inh.). Box plots show the 25%–75% interquartile range, with whiskers representing minimum and maximum values, and the median indicated. Statistical analysis was performed using the Kruskal–Wallis test with Dunn's corrections (A, D) and the Mann–Whitney U test (B, F). *: p<0.05; **: p<0.01; ***: p<0.001.

**FIGURE 3:**
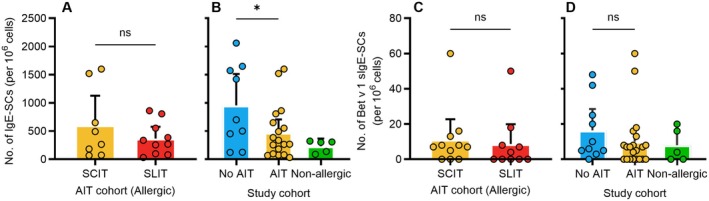
IgE‐secreting B cells as measured with ELISpot. IgE‐secreting B cells (A, B) of Bet v 1‐specific sIgE‐secreting B cells (C, D) were analysed from PBMCs as a function of allergy and allergen immunotherapy (AIT). IgE‐SC (A) or sIgE‐SC (C) frequencies in the SCIT and SLIT allergic cohorts. IgE‐SC (B) or sIgE‐SC (D) frequencies with data pooled from SCIT and SLIT (AIT) versus no AIT and non‐allergic. Means and 95% confidence intervals are shown. Differences were measured using unpaired, two‐tailed student‐*t* tests. *:p<0.05; ns: not significant.

No differences were observed in total IgE (tIgE) levels between allergic and non‐allergic participants but *Bet v 1*‐specific IgE (sIgE) levels were elevated in allergic individuals compared to non‐allergic controls (Figure 2A). Allergic individuals receiving AIT, but not the “No AIT” group, exhibited significantly higher sIgG and sIgG4 levels than those non‐allergic individuals. SCIT was also associated with an increased IgG4/IgG ratio, though no significant differences were observed in IgG/IgE or IgG4/IgE ratios (Figure 2B). ELISpot analysis revealed a high frequency of IgE‐secreting memory B cells in AIT‐treated individuals, with no difference between SCIT and SLIT patients (Figure 3A,B), but only low frequencies of Bet v 1‐specific IgE secreting B cells (Figure 3C,D). AIT‐naïve patients had an average number of 855 cells/10^6^ (SD = 722), SCIT patients had an average of 490 cells/10^6^ (SD = 538), SLIT patients 399 cells/10^6^ (SD = 314), and non‐allergic control patients 237 cells/10^6^ (SD = 109). The distribution of IgE‐SCs revealed two groups of patients according to the number of IgE‐secreting B cells. High numbers of IgE‐SCs were determined in samples no. 2, 4, 9, and 13 in the AIT‐naïve group, samples no. 16 and 17 in the SCIT group, and samples no. 27 and 36 in the SLIT group. A significant difference was seen between AIT and AIT‐naïve allergic patients (Student's *t*‐test, *p* < 0.05). These data confirm that AIT reduces the frequency of IgE memory responses while increasing levels of potentially protective IgG/IgG4 antibodies, consistent with previous findings [18, 19]. No correlation between tIgE in serum and IgE‐secreting B cells was observed (data not shown).

### 3.3 Functional Neutralisation of Allergen by AIT‐Induced IgG

To assess *Bet v 1*‐specific basophil activation, a customised RBL cell assay was developed using monoclonal anti‐*Bet v 1* IgEs for sensitisation. Three anti‐*Bet v 1* IgEs were successfully generated, and allergen‐specific, dose‐dependent degranulation was confirmed (Figure S1). We evaluated the inhibitory capacity of patient sera on RBL cell degranulation. Sera from AIT‐treated individuals and monoclonal anti‐*Bet v 1* IgG served as test and positive controls, respectively, both showing dose‐dependent inhibition of degranulation (Figure 2C). Sera from SLIT patients at a 1:40 dilution and anti‐*Bet v 1* IgG at 0.075 μg/mL reduced degranulation by over 50%. Further, serum dilutions of 1:10 and 1:20 inhibited degranulation by 97% and 94%, respectively. Overall, inhibition was significantly greater with AIT sera (SCIT: median 66%, IQR 0%–92%; SLIT: median 44%, IQR 13%–94%) compared to non‐AIT allergic (0%, IQR 0%–6%) and non‐allergic controls (0%, IQR 0%–2%) (Figure 2D). However, this inhibitory effect was observed in 14 out of 20 (70%) AIT‐treated patients (Table S1). A positive correlation was identified between degranulation inhibition and IgG/IgG4 antibody levels (Figure 2E). Sera that effectively inhibited degranulation contained significantly higher IgG and IgG4 levels than non‐inhibitory sera (Figure 2F).

### 3.4 AIT Promotes Affinity Maturation of Bet v 1‐Specific IgG‐Secreting B Cells

**FIGURE 4:**
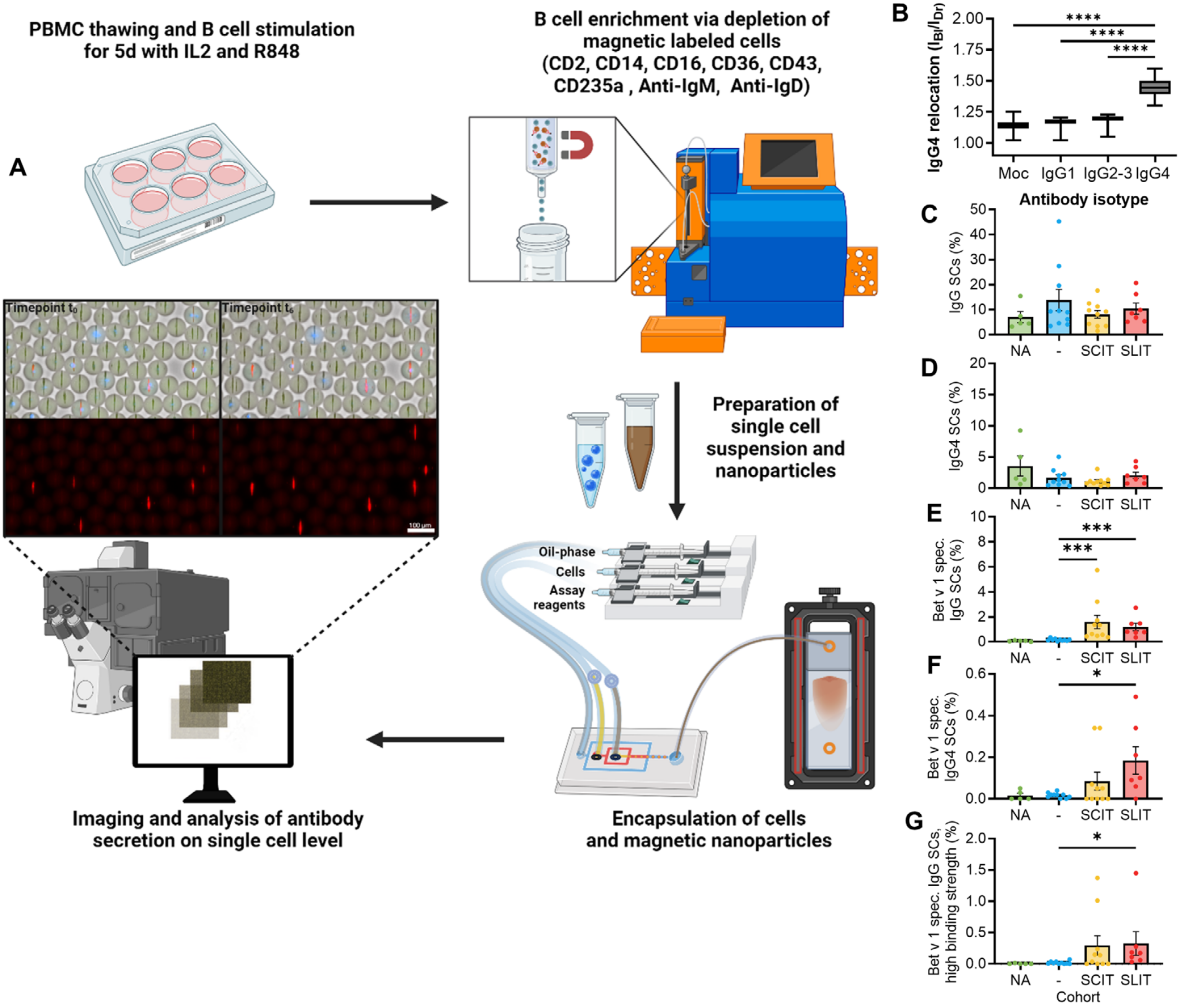
DropMap single‐cell analysis of the antibody repertoire in allergic patients. (A) Stimulation and enrichment of the IgG‐switched B cells followed by encapsulation in nanodroplets, the transfer to observation chambers, and the subsequent measurement with optical analysis. Scheme created with Biorender. (B) FITC beadline relocation measured for the four IgG isotypes as well as a blank measurement to confirm the specificity of the measuring system. Frequency of total IgG (C), total IgG4 (D), *Bet v 1*‐specific IgG (E), and *Bet v 1*‐specific IgG4 (F) secreting cells (SCs) among allergic untreated (−), allergic treated (SCIT and SLIT), and non‐allergic patients (NA). (G) Frequency of *Bet v 1*‐specific IgG‐SCs with high binding strength in untreated (No AIT) and treated (AIT) allergic patients. Each dot represents one patient sample. Histograms show mean ± SEM. Statistical analysis was performed using Kruskal–Wallis test (B–F) and Mann–Whitney U test (G). *: p<0.05; **: p<0.01; ***: p<0.001; p<0.0001.

Peripheral blood mononuclear cells (PBMCs) from study participants were analysed using DropMap technology (Figure 4A), allowing to study the secreted antibodies on the single‐cell/single‐antibody level. Two samples (#25 and #37) were excluded due to insufficient numbers of secreting cells (SCs). Calibration curves for IgG and IgG4 were successfully established with an *R* ^2^ value of 0.87 and 0.79, respectively, allowing to link measured signals to a secreted concentration (Figure S2A,B). The measurements of all four isotypes (IgG1‐4) confirmed the specificity of the anti‐human IgG4 probe displayed the assay's specificity for IgG and IgG4, respectively (Figure 4B). To distinguish different affinities, the affinity of the anti‐*Bet v 1*‐specific murine IgG1 was determined as *K*_D_ equals 6.89 × 10^−10^ for the natural TRITC labelled *Bet v 1* protein via SPR (Figure S2C). The affinity of the unlabelled natural and unlabelled recombinant *Bet v 1* proteins showed similar affinities (9.14 × 10^−10^ and 5.62 × 10^−10^) indicating no affinity differences due to the labelling. The same antibody was also introduced into the droplets and a slope of 0.1142 was determined via DropMap calibration (Figure S2D). This slope has been shown to correlate with K_D_ in previous experiments using different antigens [20], and this value was further used as a threshold to classify secreted *Bet v 1* specific antibodies as high‐ or low‐affinity, i.e., exceeding the affinity of the commercial Bet v1 antibody or not.

Across all groups (non‐allergic, allergic, SCIT, SLIT), comparable frequencies of IgG (Figure 4C) and IgG4 SCs (Figure 4D) were detected. However, PBMCs from AIT‐treated individuals exhibited higher numbers of *Bet v 1*‐specific IgG‐secreting B cells (Figure 4E), significantly increased frequencies of allergen‐specific IgG4‐SCs (Figure 4F), and antibodies with greater binding strength to *Bet v 1* (Figure 4G) compared to untreated individuals.

### 3.5 IgG‐Epitope Mapping on Bet v 1 Reveals Distinct Binding Patterns

**FIGURE 5:**
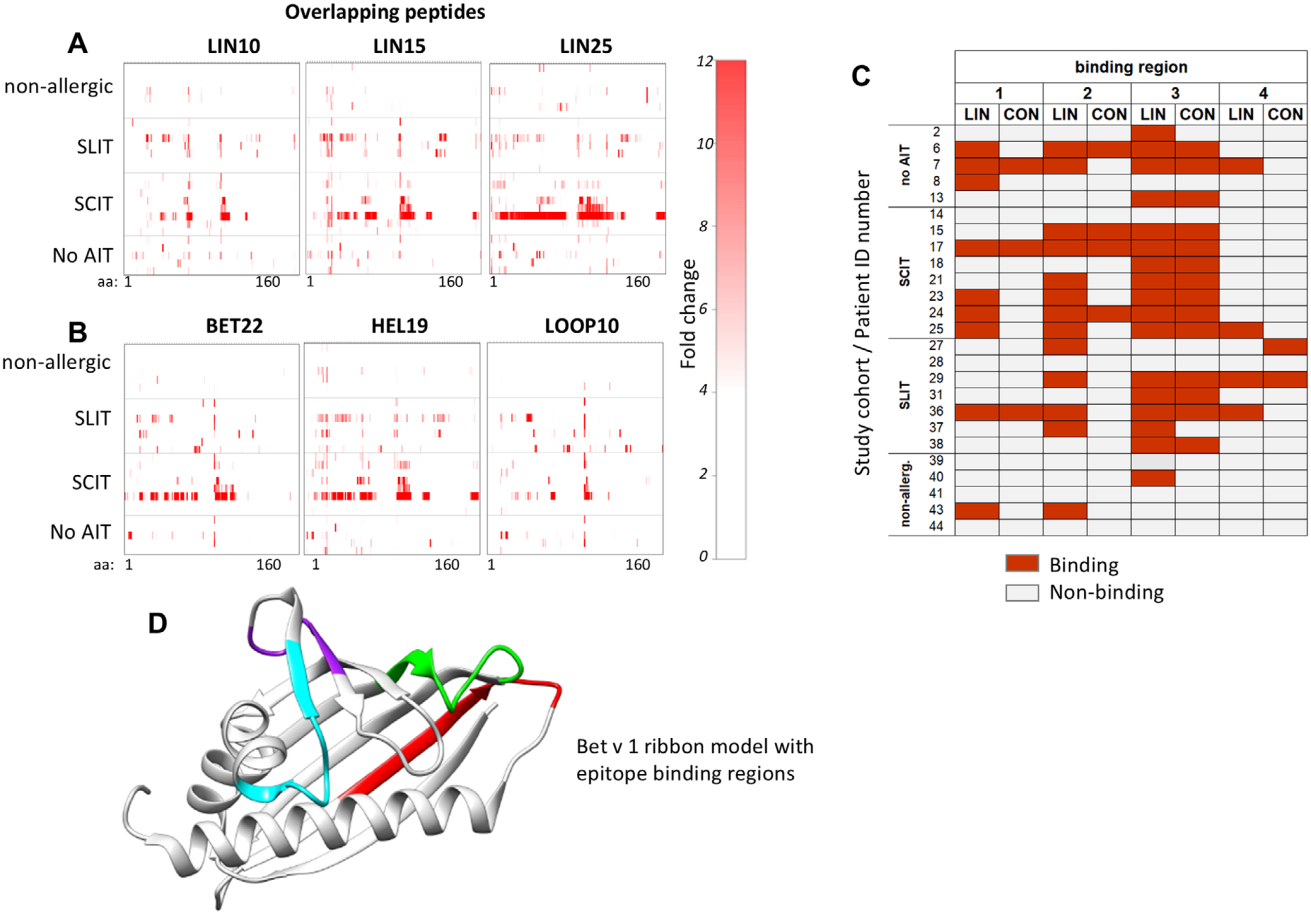
Epitope mapping of sera from birch pollen‐allergic patients using overlapping Bet v 1 peptides. (A) Heat map of IgG binding, generated using linear peptides of 10 (LIN10), 15 (LIN15), and 25 (LIN25) amino acids. (B) Heat map of IgG binding using conformational β‐sheet peptide mimic of 22 amino acids (BET22), alpha‐helix of 19 amino acids (HEL19), and loop of 10 amino acids (LOOP10). All heat maps are organised with *Bet v 1* sequence on *x* axis and patient cohort on *y*‐axis. The colour scale represents the fold change of binding to peptide sequences as compared to negative controls. (C) Summary of IgG binding of individual patient sera to putative linear and conformational epitopes in the defined binding regions 1–4. (D) Structural model of *Bet v 1* highlighting putative epitope regions. The *Bet v 1* model is shown in ribbon format, with the identified binding regions colour‐coded: Cyan (34‐VAPQAISSVE‐43), purple (49‐GGPGTIKK‐56), green (86‐VIEGGPIGDT‐95), and red (117‐ISNKYHTKGD‐126).

IgG epitope mapping was performed on IgG‐purified sera from 20 selected birch‐pollen allergic patients (5 untreated, 8 SCIT, 7 SLIT) and 5 non‐allergic control patients in collaboration. Overlapping peptide arrays (Table S2) were used for linear and conformational mapping of IgG from patient sera on *Bet v 1*, a 18kD protein of 160 amino acids (Table S3). Purified IgG from birch pollen–allergic patients receiving SCIT or SLIT displayed stronger binding to *Bet v 1* peptide libraries compared to IgG from untreated allergic individuals (Figure 5A,B, Figure S3). Four main epitope binding regions were identified (Figure 5C,D):

- *Region 1* (*aa34‐43*): Located between α2 and β2, recognised by 50% (4 out of 8) of the SCIT sera and 14% (1 out of 7) of the SLIT sera (#36), with minimal recognition by non‐allergic individuals.
- *Region 2* (*aa48‐55*): Positioned in the β‐sheet region, identified by 75% (6 out of 8) of the SCIT and 57% (4 out of 7) of the SLIT sera, with stronger binding by SCIT sera.
- *Region 3* (*aa86‐95*): Also in the β‐sheet, bound by 60% (3 out of 5) of untreated, 88% (7 out of 8) of SCIT, and 57% (4 out of 7) of SLIT sera. Conformational recognition was common.
- *Region 4* (*aa117‐126*): Spanning the final β7 and the C‐terminal α3, exclusively recognised by SLIT sera (43%; 3 out of 7). A linear peptide of 15 amino acids within the N‐terminal region (aa111‐125) was uniquely recognised by SLIT patient sera. One SLIT serum (#27) out of seven (14%) recognised a looped peptide spanning through the C‐terminal region (aa121‐132).

Additionally, some SCIT and SLIT sera bound peptides in the far C‐terminal region (no‐AIT: 20%; SCIT: 50%; SLIT: 57%), with several samples exhibiting unique individual‐specific binding patterns (Table S4).

### 3.6 High Antibody Levels and IgG Binding to Region 1 Correlate With Improved Clinical Outcomes

**FIGURE 6:**
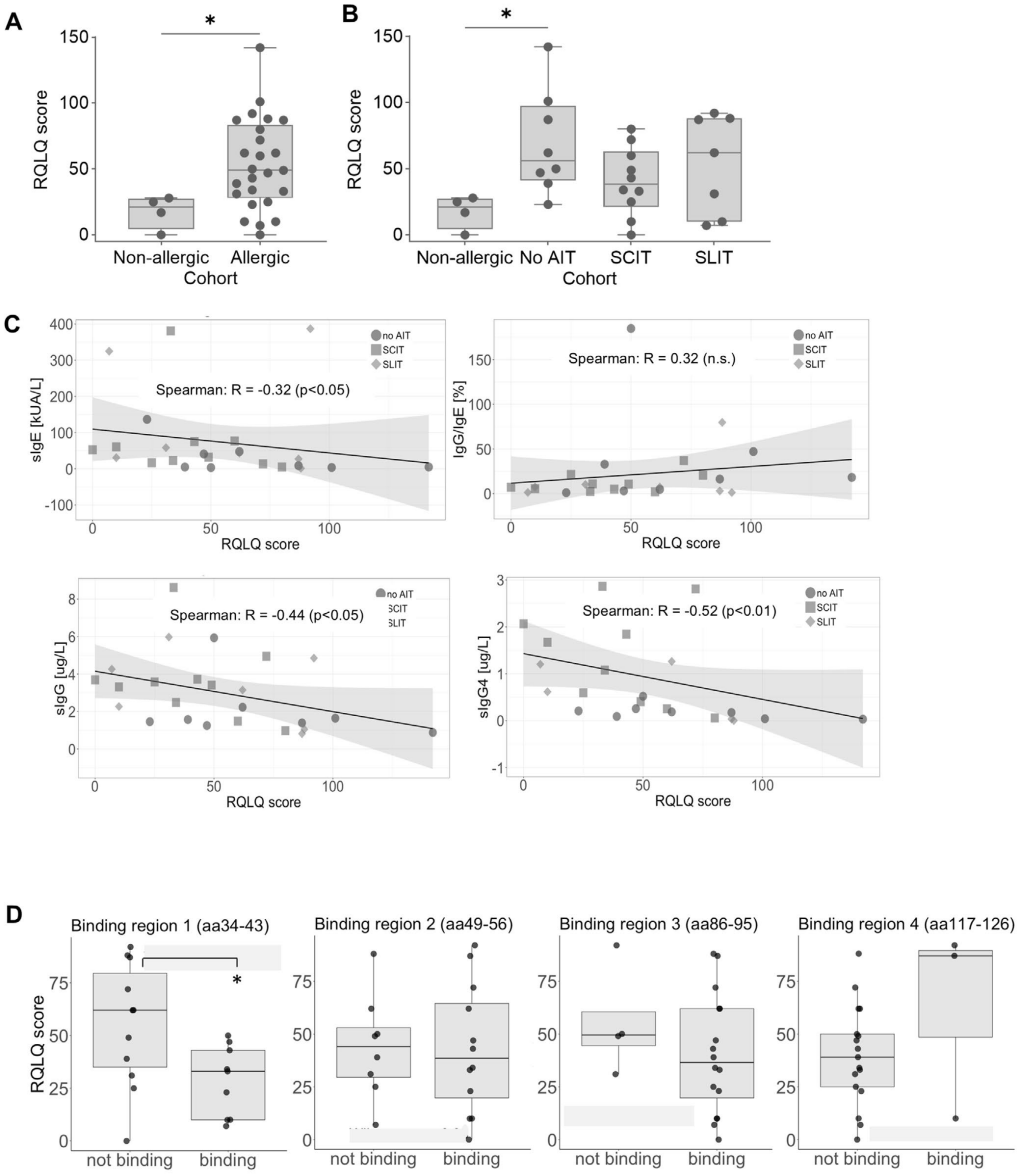
Rhinitis‐related Quality of Life (RQLQ) and correlation with serum antibodies. (A‐B) RQLQ scores comparing allergic and non‐allergic patients (A) or AIT status (SCIT, SLIT, or none) (B). A total of 29 out of 35 study participants returned RQLQ score sheets. Statistical analysis was performed using the Mann–Whitney U test for Non‐allergic vs. No AIT (A) and the Kruskal‐Wallis test for No AIT vs. SCIT vs. SLIT (B). (C) Spearman correlations between serum levels of specific IgE (sIgE), IgG (sIgG), IgG4 (sIgG4), or the sIgG/sIgE ratio and RQLQ scores in birch pollen‐allergic patients. (D) RQLQ scores in birch pollen‐allergic patients as a function of binding to linear and/or conformational epitopes in IgG‐binding regions 1, 2, 3, and 4 of *Bet v 1*. Statistical analysis was performed using the Mann–Whitney *U* test.

A RQLQ was completed by 29 participants (Table S1). Allergic individuals reported significantly higher RQLQ scores compared to non‐allergic controls (Figure 6A), with no significant difference between allergic patients with and without AIT (Figure 6B). Negative correlations were observed between sIgE, sIgG, and sIgG4 levels and RQLQ scores in allergic patients (Figure 6C), but no correlation was found for sIgG/sIgE ratios. Total IgE levels did not correlate with RQLQ scores. Notably, patients with IgG binding to Region 1 exhibited significantly lower RQLQ scores, indicating improved quality of life (Figure 6D). No significant associations were observed between RQLQ scores and binding to Regions 2–4.

### 3.7 Bet v 1 Epitope‐Based Synthetic Peptides Are Hypoallergenic In Vitro

Four peptide sequences of each 25–30 amino acids were designed based on the identified *Bet v 1* epitopes (Table S5) and synthesised with N‐terminal amidation and C‐terminal acetylation. LC–MS confirmed their molecular weights (Figure S4), and purities exceeded 95% (Figure S5). Two additional peptides (#5–6) incorporated known IgE epitopes. Peptide #5 (30 amino acids) included key surface patches (aa49–58, aa73–88, and aa88–103) known to represent major IgE‐binding sites [21, 22, 23, 24]. Previous work has shown that this region allows the simultaneous binding of multiple IgE antibodies and that monoclonal antibodies directed against it can inhibit allergic responses [22, 25]. Notably, IgG‐binding regions 2 and 3 partially overlapped with this IgE‐reactive surface. Peptide #6 (34 amino acids) was designed to extend region 3 and cover an additional IgE‐binding site [24]. Because we used overlapping peptides for epitope mapping, discontinuous epitopes like the previously described BV16 p‐loop epitope [26] could not be mapped or confirmed.

**FIGURE 7:**
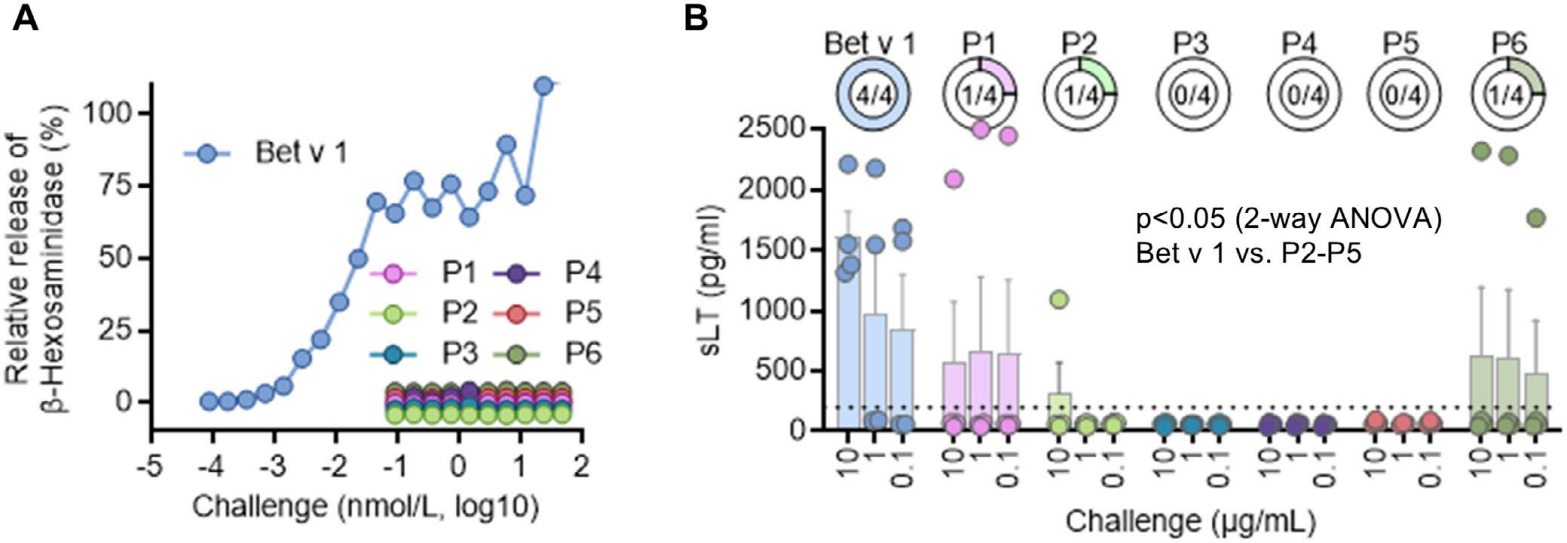
β‐Hexosaminidase and sLT release from sensitised cells in response to antigen stimulation. (A) β‐Hexosaminidase release from sensitised RBL cells upon challenge with *Bet v 1* or peptides (Pep1–6). (B) CAST ELISA performed on leukocytes from birch pollen‐allergic patients (*n* = 4). Stimulation was conducted at three different concentrations (10, 1, and 0.1 μg/mL), and sLT release was measured (cut‐off set at 200 pg/mL). The donut charts in the upper part of the graph indicate the number of individuals whose cells exhibited sLT release > 200 pg/mL following antigen stimulation. Data are presented as means ± SD.

Despite the heterogeneity in IgG binding, all six peptides were tested for allergenicity using sensitised RBL cells. Unlike the full *Bet v 1* protein, the peptides did not induce degranulation or sLT release even at 10 to 100,000‐fold higher concentrations (Figure 7A). In addition, a cellular antigen stimulation test using leukocytes from four *Bet v 1*‐allergic donors confirmed these findings. While *Bet v 1* triggered strong leukocyte degranulation, peptides 1, 2, and 6 caused minimal degranulation in only one donor, and peptides 3, 4, and 5 did not elicit any response (Figure 7B).

## 4 Discussion

While AIT has been proven effective for many IgE‐mediated allergies [1, 2], a substantial proportion of allergic patients either fail to initiate or do not complete AIT [16, 17]. A broader application of AIT is hindered by the risk of allergic side effects, including systemic anaphylaxis, and by inconsistent treatment responses. The development of safer and more effective AIT formulations—particularly those that minimize IgE reactivity while promoting allergen‐neutralising IgG—could help overcome this therapeutic gap.

In this study, we used the major birch pollen allergen *Bet v 1* as a model to identify IgG‐binding epitopes in allergic individuals, leveraging patient‐derived data to design candidate peptides for safer AIT. Using sera from both AIT‐treated and untreated individuals, we identified four distinct IgG‐binding regions commonly recognised across allergic patients, irrespective of their AIT status, and spanning both linear and conformational epitopes. Binding region 1 (aa34–43) was associated with improved clinical symptoms, as measured by RQLQ scores, suggesting potential functional relevance. IgG binding to other regions also differed by AIT modality, underscoring variability in immune targeting across individuals.

Interestingly, 80% of the patients receiving AIT displayed IgG binding to linear epitopes in region 3 (aa86–95), and most of these also recognised conformational epitopes in the same region. Binding region 4 (aa117–126) was predominantly recognised by sera from SLIT‐treated patients. On the individual level, one SCIT sample exhibited strong IgG binding to regions 1–3, while one SLIT sample bound to all four regions. Both sera came from patients with high RQLQ scores and demonstrated effective inhibition of allergen‐induced basophil degranulation in vitro. However, IgG binding patterns were highly patient‐specific and extended beyond the four consensus regions, highlighting the need for personalised strategies in AIT peptide design to maximise patient coverage.

To explore the clinical potential of these epitopes, four peptides (#1–4, 25–30 amino acids) were designed based on the identified IgG‐binding regions. Two additional peptides (#5–6) incorporated known IgE epitopes. Peptide #5 included key surface patches (aa49–58, aa73–88, and aa88–103) known to represent major IgE‐binding sites [21, 22, 23, 24]. Previous work has shown that this region allows the simultaneous binding of multiple IgE antibodies and that monoclonal antibodies directed against it can inhibit allergic responses [22, 25]. Notably, IgG‐binding regions 2 and 3 partially overlapped with this IgE‐reactive surface. Peptide #6 was designed to extend region 3 and cover an additional IgE‐binding site [24]. Because we used overlapping peptides for epitope mapping, discontinuous epitopes like the previously described BV16 p‐loop epitope [26] could not be mapped or confirmed. Despite the heterogeneity in IgG binding, all six peptides exhibited markedly reduced allergenic activity in vitro, failing to induce basophil degranulation or sLT release even at supraphysiologic concentrations. However, the two peptides overlapping with known IgE epitopes [21, 24], raise concerns that not all IgG‐targeted sequences are necessarily safe. Peptide #5, for instance, overlapped with regions known to bind multiple IgE clones [22]. This underlines the importance of balancing IgG immunogenicity with IgE safety profiles. This hypoallergenicity is particularly important given the dose‐limiting toxicity of current allergen extract‐based therapies and underscores the potential safety advantage of such peptides over full‐length *Bet v 1* or extract‐based AIT.

Designing AIT peptides exclusively based on IgG‐binding epitopes, however, is not without risks. Some IgG antibodies may lack allergen‐neutralising function, or worse, enhance allergen presentation and IgE engagement [27, 28]. This may explain why high IgG titres do not always correlate with clinical improvement post‐AIT [29]. Conversely, targeting IgE‐binding epitopes can inadvertently activate effector cells if peptide structure permits FcεR cross‐linking, particularly when these peptides are fused to carrier molecules to boost immunogenicity, potentially heightening allergenic activity.

Understanding the contribution of continuous, conformational, and discontinuous epitopes is essential for inducing clinically relevant, blocking IgG antibodies, and a key question is whether AIT‐induced IgG shares clonal origin or epitope specificity with IgE [15]. Some studies have reported significant epitope overlap between allergen‐specific IgE and IgG4 after AIT [15, 30, 31], whereas others describe polyclonal expansion and the emergence of novel IgG specificities during therapy [32]. These apparent discrepancies may reflect differences in treatment modalities (conventional SCIT or SLIT versus more experimental oral or epicutaneous AIT) and patient populations (children, adolescents, or adults), which may be expected to influence the specificity, breadth, kinetics of IgG and IgE, the stimulation of effector and regulatory T cells, and finally the induction of tolerance [33]. Moreover, the nature and function of naturally occurring IgG antibodies remain unclear. *Bet v 1*‐specific IgG antibodies from non‐allergic individuals have been shown to preferentially bind linear epitopes, while IgE antibodies target conformational epitopes [34]. Our findings align with this, and our data suggest that effective AIT peptide design must account for epitope structure—linear, conformational, and discontinuous—and support further investigation of epitope overlap between IgE and IgG.

Using DropMap single‐cell microfluidics, we found that AIT enhances the number and affinity of *Bet v 1*‐specific IgG‐secreting B cells, suggesting clonal maturation within the memory compartment. Interestingly, while total IgG4 levels increased, the isotype composition of individual B‐cell responses remained largely stable. These findings highlight the importance of affinity maturation, rather than isotype switching alone, in generating protective antibody responses. Moreover, our data indicate that naturally occurring IgG antibodies in non‐allergic individuals preferentially target linear epitopes with low functional activity. In contrast, AIT‐treated patients exhibit higher‐affinity IgG responses with superior capacity to inhibit effector cell activation—supporting the functional relevance of treatment‐induced IgG. This study also demonstrated the utility of DropMap technology in tracking affinity maturation at the single B‐cell level. This approach holds promise for a more precise characterisation of B‐cell responses to AIT and potentially guiding the development of improved therapeutic strategies.

Since our strategy for designing AIT peptides was based on mapping the IgG‐binding epitopes of an allergen, here *Bet v 1*, using patient‐derived immune profiles, the resulting peptides may possibly not contain any T‐cell epitopes. Hence, to ensure activation of T‐cell help for B‐cell‐mediated IgG responses, the designed peptides could be formulated together with a universal T‐cell epitope. Although the *Bet v 1*‐derived peptides described here do not contain allergen‐specific T‐cell epitopes and are not expected to stimulate allergen‐specific T cells, this limitation can be addressed by incorporating a well‐characterised, promiscuous CD4 T‐cell epitope such as one derived from tetanus toxoid [35] cytomegalovirus [36], or SARS‐CoV‐2 [37] These epitopes are recognised by memory T cells in most individuals due to widespread vaccination or natural exposure, and have been successfully used in other vaccine platforms to provide T‐cell help independently of the antigen‐specific T cell repertoire [35, 38, 39, 40]. This strategy offers a rational path forward for driving affinity‐matured, protective IgG responses without engaging intrinsic allergen‐specific T cells.

Collectively, this study introduces a patient‐centred framework for rational AIT peptide design based on mapped IgG epitopes. By integrating immune profiling, peptide engineering, and in vitro functional assays, we identify hypoallergenic peptide candidates that warrant further preclinical and clinical evaluation. This approach may serve as a blueprint for epitope‐based therapies beyond allergy, offering a strategy for selectively engaging protective immune responses while avoiding immunopathology.

This study is limited by its in vitro nature and sample size, which may not capture the full variability of allergen‐specific immune responses across diverse populations. While the mapped IgG‐binding epitopes were derived from clinically relevant patient samples and correlated with functional activity, their immunogenicity and safety have not yet been evaluated in vivo. Additionally, the reliance on linear peptide libraries may underrepresent conformational or discontinuous epitopes critical for functional IgG responses. Another limitation is the use of an artificial system for basophil activation assays, where a mixture of three monoclonal IgEs was employed to sensitise effector cells. While this model allows for controlled assessment of allergenic potential, it does not fully recapitulate the polyclonal and highly individualised nature of IgE repertoires found in allergic patients, potentially underestimating or misrepresenting the breadth of IgE‐mediated responses. Further studies are needed to assess the therapeutic potential of the candidate peptides in animals in allergic individuals, including clinical safety assessments (e.g., skin prick testing) and immunogenicity profiling in larger, diverse cohorts. Evaluating whether these peptides can elicit long‐lived, allergen‐neutralising IgG responses without triggering IgE‐mediated activation will be essential for translation into clinical immunotherapy.

## Author Contributions

Conceptualization: L.Š., C.S., K.E., P.J. Investigation: L.Š., A.S., M.P., D.M., T.J. Medical advisors: C.C.V.L., T.M.K. Scientific advisor: R.C. Fund raising: T.M.K., P.J. Writing, original draft: L.Š., A.S., M.P., P.J. Writing – review and editing: L.Š., A.S., C.S., K.E., P.J. All authors approved the manuscript before submission.

## Funding

This project is funded by the University of Zurich and the University Hospital Zurich. MP were in part paid by funds from Innosuisse (25866.2 PFLS‐LS). The project received financial support from Foundation for Research in Science and the Humanities at the University of Zurich (STEB‐2022‐002). DM was in part paid by funds from Swiss National Science Foundation (#407940_206399).

## Conflicts of Interest

The authors declare no conflicts of interest.

## Supporting information


**Data S1:** cea70276‐sup‐0001‐Supinfo.docx.

## Data Availability

The data that support the findings of this study are available from the corresponding author upon reasonable request.
