# Supplementary material for: Patient‐Derived IgG Epitope Mapping of Bet v 1 Reveals Hypoallergenic Peptide Candidates for Safe and Next‐Generation Allergen Immunotherapy
Source: Clin Exp Allergy. 2026 Mar 10;56(8):881–92. doi: 10.1111/cea.70276 (PMC13429318; doi:10.1111/cea.70276)
Supplement: Supplementary file 1 — Data S1: cea70276‐sup‐0001‐Supinfo.docx. [file CEA-56-881-s001.docx]

Patient-derived IgG epitope mapping of Bet v 1 reveals hypoallergenic peptide candidates for safe and next-generation allergen immunotherapy

Lara Šošić ^[a]^, Marta Paolucci ^[a]^, Alessandro Streuli ^[b], [c]^, David A. Melillo ^[a]^, Tianchi Jiang ^[d]^, Raffaela Campana ^[d]^, Claudia C.V. Lang ^[a], [e]^, Thomas M. Kündig ^[a], [e]^, Christian Steuer ^[c]^, Klaus Eyer ^[b], [f]^, and Pål Johansen ^[a], [e]^

[a] Department of Dermatology, University of Zurich, Wagistrasse 18, 8952 Schlieren, Switzerland

[b] Laboratory for Functional Immune Repertoire Analysis, Institute of Pharmaceutical Sciences, Department of Chemistry and Applied Biosciences, ETH Zurich, Vladimir-Prelog-Weg 1-5/10, 8093 Zurich, Switzerland

[c] Laboratory for Pharmaceutical Analytics, Institute of Pharmaceutical Sciences, Department of Chemistry and Applied Biosciences, ETH Zurich, Vladimir-Prelog-Weg 1-5/10, 8093 Zurich, Switzerland

[d] Division of Immunopathology, Department of Pathophysiology and Allergy Research, Center for Pathophysiology, Infectiology and Immunology, Medical University of Vienna, Kinderspitalgasse 15, 1090 Vienna, Austria

[e] Department of Dermatology, University of Zurich, Raemistrasse 100, 8091 Zurich, Switzerland

[f] Department of Biomedicine, Aarhus University, Bartholins Allé 6, Building 1242, 8000 Aarhus, Denmark

Experimental procedures

Rhinitis quality of life questionnaire (RQLQ)

Between 12–16 months after blood collection (April 1, 2022), the 35 study participants (**Suppl. Table S1**) received a standardised paper version of the RQLQ [1]. They assessed rhinitis-related quality of life during the 2022 birch pollen season, specifically referencing the seven days prior to completion.

Blood processing and analysis

PBMCs were isolated via Ficoll-Paque density gradient centrifugation and stored in 10% DMSO/fetal calf serum at -80 °C. Sera were prepared from clotted blood and stored at -20°C for analysis of total IgE (tIgE, kUA/L), *Bet v 1*-specific IgE (sIgE, kUA/L), *Bet v 1*-specific IgG (sIgG, µg/L), and IgG4 (sIgG4, µg/L) using ImmunoCAP on Phadia 250 (ThermoFisher Scientific).

Secretion and antigen specificity analysis of IgE-secreting B cells in ELISpot

The ELISpot was performed on membrane plates coated either with *Bet v 1* (5 μg/mL) or anti-IgE antibody (1 μg/mL). PBMCs were then added at 1.0×10^6^ cells/mL in supplemented cell culture medium (RPMI 1640, glutamine, 1% Pen/Strep and 10% FCS, all ThermoFisher) and cultured with the addition of 10 ng/mL IL-4 and 1 μg/mL anti-CD40 (Biolegend) for five days. The ELISpot was developed, and the frequencies of IgE-secreting B cells and antigen-specific IgE-secreting B cells were measured using the Human IgE (ALP) ELISpot Flex Kit according to the supplier’s instructions (Mabtech). Images were recorded on an ELISpot/FluoroSpot Reader (Autoimmun Diagnostika GMBH). The results are shown as number of spots per 1×10^6^ cells.

DropMap single-cell analysis of IgG-secreting memory B cells

PBMCs were stimulated with 40 ng/mL IL-2 and 1 μg/mL R848 (Miltenyi Biotec) for five days. Switched memory B cells were purified using the Memory B cell isolation kit (Miltenyi Biotec), stained with 5 µM CellTrace violet (ThermoFisher), and diluted to 7.5–10 million/mL, achieving 0.3–0.4 cells/droplet. Single-cell analysis was conducted via DropMap [2, 3] (**Fig. 4**). In short, magnetic nanoparticles (streptavidin plus, 300 nm diameter, Ademtech) were coated with CaptureSelect™ Biotin Anti-IgG-Fc (ThermoFisher). IgG secretion was measured with Alexa 647 AffiniPure F(ab')₂ Fragment Goat Anti-Human IgG, Fcγ fragment specific (Jackson Immuno, 75 nM in-drop concentration), FITC Mouse anti-human IgG4 Fc (SouthernBiotech, 75 nM in-drop concentration) and labelled antigen (natural Bet v1, Inbio, 15 nM in-drop concentration, labelled with AlexaFluor 555, Thermo Fisher). Droplets of 65 pL in volume were produced using hydrodynamic flow-focusing and collected in an observation chamber. For each sample, two observation chambers were measured over 90 minutes (6 time points, 15 minutes in between) simultaneously. For imaging, the filled chamber was mounted on an epifluorescence microscope (TI2 Eclipse, Nikon). Images were analysed using a custom MATLAB script (Mathworks, version R2022B) [2-5].

The DropMap analysis was calibrated and validated (**Fig. 4B-F)**. An antigen calibration curve was generated by coating nanoparticles with anti-murine capture IgG (mouse V_H_H capture biotin anti-Lc kappa, Thermo Scientific) and measuring increasing concentration with anti-*Bet v 1*-specific murine IgG1 (2.5 – 100 nM, MA-5H8SB, Inbio) together with constant concentration of fluorescently-labelled *Bet v 1* antigen (15nM) and fluorescently-labelled anti-IgG (75nM, goat anti-mouse IgG, Fc-AF647, Lot. J331699450, USA). The relocation of the antigen was plotted against the relocation of the antibody and linearly fitted (One-phase association, GraphPad Prism). Measurements were performed in triplets. The slope of the curve was used to estimate the binding-strength of secreted antibodies of human samples against the antigen. For antibody secretion and isotope-specificity, IgG and IgG4 calibration curves were first generated using 0 – 100 nM human IgG4 isotype control and prepared magnetic nanoparticles. The specificity of the IgG4 detection was verified by parallel testing with human IgG1, IgG2 and IgG3 isotypes. For further analysis, the total IgG calibration curve was used for quantification, and the IgG4 calibration curve was used as an indicator for the isotype group.

Classification of SCs in IgG-SCs, IgG4-SCs, and B cells with *Bet v 1* specific antibodies was performed as described [3]. In short, the beadline relocation for each channel was determined, except for the DAPI channel where the overall DAPI signal for cell detection was measured for every droplet. IgG-SCs were classified in IgG4 SCs when having at least one analyte relocation in the FITC-channel above LOD and B cells with *Bet v 1* specific antibodies when displaying one analyte relocation in the TRITC-channel above LOD, having a positive slope over time for the antigen channel and showing a binding strength curve that is significantly different from 0. Each B cell with *Bet v 1* specific antibodies was further classified as high-binding (slope > 0.1142) or low-binding (slope < 0.1142). Patient samples with less than 100 SCs (#25 and #37) were excluded from further analysis [2-4]. The number of cells found in each category was normalised to the amount of total IgG-SCs.

IgG epitope mapping of *Bet v 1*

Serum IgG was purified from patient sera using NAb protein G spin columns (Thermo Fisher Scientific), dialysed with slide-A-lyzer dialysis cassettes (Thermo Fisher Scientific), and concentrated with 100 kDa Amicon centrifugal filter units (Merck). IgG epitope mapping was performed on IgG-purified sera from 20 selected birch-pollen allergic patients (5 untreated, 8 SCIT, 7 SLIT) and 5 non-allergic control patients in collaboration with Biosynth (Lelystad, the Netherlands). Overlapping peptide arrays (**Suppl. Table S2**) were used for linear and conformational mapping of IgG from patient sera on *Bet v 1*, a 18kD protein of 160 amino acids (**Suppl. Table S3**). For conformational epitope mapping, the Chemically Linked Peptides on Scaffolds (CLIPS) technology was used [6]. The linear and CLIPS peptides were synthesised using standard Fmoc-chemistry and deprotected using trifluoric acid with scavengers. The binding of serum antibody to each peptide was tested in a peptide ELISA. The 455-well credit card format polypropylene cards containing the covalently linked peptides were incubated with primary antibody solution. After washing, the peptides were incubated with a 1/1000 dilution of antibody peroxidase conjugate for one hour at 25°C. After a washing step, peroxidase substrate 2,2’-azino-di-3-ethylbenzthiazoline sulfonate (ABTS) and 2 μl of 3% H2O2 were added. After one hour, the colour development was quantified with a charge coupled device (CCD) [7]. The results were visualised using bar or line plot showing the relative binding intensity per peptide, as well as with epitope tables and heat maps. The heat maps incorporate results from duplicate screenings of each sample. Individual binding peptides were highlighted when values were at least 4 times higher than the background value, which was defined as the 10th percentile for the specific peptide mimic.

In addition, individual overlays were made of sample and negative controls to identify specific binding peaks for each individual sample. Peaks were defined as values at least 2.5-fold higher than the background value, which was defined as the intensity value of the same peptide in the negative control screening. The final putative epitopes were defined based on the combined results of the heat maps and the sample/isotype overlays. Putative core epitopes were identified as adjacent peptides located within the regions identified by the heat maps, with similar or a maximum of 30% lower intensity compared to the top peak within a binding region. Furthermore, if peaks were well-defined and contained multiple overlapping peptides, lower intensity peaks may also be indicated. For a comprehensive evaluation of the putative epitopes, information was extracted from multiple mimic types. In general, peptides mimics with the highest binding were prioritised; if mimics showed similar binding intensities, the shorter mimic was used for epitope determination.

Production of monoclonal anti-*Bet v 1* IgE and IgG

Three monoclonal anti-*Bet v 1* IgE and three IgG antibodies were transiently expressed in HEK293 cells. Antibody sequences were based on commercial monoclonal human IgG antibodies targeting non-overlapping *Bet v 1* epitopes [8-10], cloned into pTwist-CMV-BetaGlobin-WPRE-Neo vectors (Twist Bioscience), and transformed into DH5α *E. coli* (New England Biolabs). Plasmids were extracted, purified (Zymo Research), and quantified. HEK293 cells were transfected; heavy chains were C-tagged (EPEA sequence). Antibodies were purified by FPLC (Cytvia), quantified by Pierce BCA assay, and heterotetrameric assembly confirmed by SDS-PAGE. Binding to *Bet v 1* was assessed by ELISA (**Suppl. Fig. S1A**).

Synthesis of hypoallergenic Bet v 1 peptides

Linear peptides of 25-34 amino acids (**Suppl. Table S5**) were synthesised via automated solid-phase peptide synthesis on a PurePrep Chorus peptide synthesizer (Gyros Protein Technologies, USA) using Rink amide resin (100-200 mesh, 0.52 mmol/g loading, aaptec, USA). Synthesis was performed according to standard protocols using dimethylformamide (DMF) as solvent and swelling agent, pyrrolidine in DMF (20% v/v) as deprotection agent and a solution of 5 eq. O-(1H-6-Chlorobenzotriazole-1-yl)-1,1,3,3-tetramethyluronium hexafluorophosphate (HCTU) and 10 eq. 2,6-dimethylmorpholine (NMM) in DMF together with 5 eq. pure Fmoc protected amino acids (ProteinTechnologies, United Kingdoms) as coupling agent. Acetylation at the NH2-terminus was achieved by treatment with 20% acetic anhydride in DMF. Peptides were cleaved and fully deprotected with 95% TFA, 2.5% TIS, 2.5% nanopure water (v/v/v) [11, 12]. Peptides were washed twice with ether and purified by C18 reversed-phase flash chromatography (puriFlash XS520Plus, Interchim, France). Salt exchange was performed twice by dissolution in 10 mM HCl and subsequent freeze drying.

Mass accuracy of peptides was confirmed by LC-MS (**Suppl. Fig. S4**) and purity by RP-HPLC-UV (**Suppl. Fig. S5**) after dissolving the peptides in a 1:1 solution of acetonitrile and water with 0.1% formic acid with a concentration of 0.1 mg/mL. LC-MS analysis was performed using an LTQ-XL linear ion trap equipped with a heated ESI II source (Thermo Scientific, San Jose (CA), United States). The mass spectrometer was coupled to a Waters Acquity™ UPLC system (Milford (MA), United States). Gradient elution was done on an Zorbax Eclipse Plus C18 reversed-phase column (2.1 × 50 mm, 1.8 µm; Agilent Technologies, Santa Clara, CA) at room temperature. The mobile phase consisted of 0.1% formic acid in water (eluent A) and 0.1% formic acid in acetonitrile (eluent B). Flow rate was set to 0.5 mL/min and the injection volume to 10 µL. The used gradient was as followed: 0-2 min 5% B, 2-10 min to 70% B, 10-12 to 90% B, 12-15 min 90% B, 15-15.5 min to 5% B,15.5-20 min 5% B. The autosampler was set to 10 °C. All MS measurement was performed in positive ionisation mode. The ESI source was used in a non-heated state. Sheath gas and auxiliary gas were set to 34 and 11 arbitrary units, respectively. Source voltage was 5.00 kV; temperature of the ion transfer capillary was 275 °C. Capillary voltage was 31 V; tube lens voltage was 80 V. MS was performed in full scan mode (m/z 100-2000).

Purity control was performed using a VWR ELITE Lachrome Series LC modular system consisting of a L-2130 gradient pump, L-2200 autosampler, L-2350 column oven and a L-2455 diode array detector. OpenLab software (Version A. 04.08 - Agilent Technologies, Santa Clara, CA, USA) was used for system operation as well as peak interpretation. For chromatographic separation, an Eclipse Plus C18 column (3.0 μm × 150 mm, SN: USUXP01161, Agilent Technologies, California, United States) without a guard column was used. Mobile phases consisted of 0.1% TFA in water (eluent A) and 0.1% TFA in acetonitrile (eluent B). Flow rate was set to 1.0 mL/min and the injection volume to 4 µL. The used gradient was as followed: 0-5 min 5% B, 5-32.5 min to 60% B, 32.5-35.0 min to 95% B, 35.0-40 min 95% B, 40.0-41.0 min to 5% B, 41.0-50.0 min 5% B. The autosampler was set to 5 °C and the column oven to 30 °C. Scan settings of the diode array detector (DAD) were as follows: recording from 210 to 300 nm with individual scans at 214 and 254 nm, sampling period of 400 ms and response time of 1 second.

RBL degranulation and inhibition assays

RBL-2H3 clone 21/2C5 cells expressing human FcɛRIα (Paul-Ehrlich-Institute) were cultured in RPMI-1640 (Thermo Fisher Scientific) with 10% FCS, 1% L-glutamine, Normocin (0.9 mg/mL), and initially selected with G418 (1 mg/mL). Cells were sensitised overnight with 0.5 µg/mL monoclonal anti-*Bet v 1* IgE antibodies. Sensitisation was confirmed via anti-human IgE staining and in-house ELISA. Sensitised cells were challenged with *Bet v 1* or irrelevant allergens for one hour. β-hexosaminidase release was quantified by p-nitrophenyl-N-acetyl-β-D-glucosaminide substrate assay (Sigma), stopped with 0.2 M glycine, and OD measured at 405 nm (620 nm reference). The assay was optimised for *Bet v 1* challenge dose and IgE sensitisation dose (**Suppl. Fig. S1B-C**). To assess IgG-mediated inhibition, sensitised RBL cells were incubated with 0.01 µg/mL recombinant *Bet v 1* pre-incubated with serially diluted, heat-inactivated (56°C, 30 min) patient sera (starting dilution 1:10). *Bet v 1*-specific IgG served as control. Relative β-hexosaminidase release was calculated as (OD_sample_ – OD_spontaneous_) / OD_total_. The relative inhibition (in %) of β-hexosaminidase release after a challenge with patient serum to that of cells challenged without serum was then determined.

Peptide allergenicity assessment in human blood

Leukocytes from *Bet v 1*-allergic patients were isolated using the Bühlmann CAST ELISA kit and stimulated in vitro with 0.01–10 µg/mL *Bet v 1* protein or peptides at 37°C. After 40 minutes, leukotrienes LTC4, LTD4, and LTE4 were quantified from supernatants by CAST ELISA.

Expression and purification of recombinant Bet v 1

*Bet v 1*.0101 (GenBank: CAA33887) was cloned into the NdeI and EcoRI restriction site of plasmid pET-17b (Novagen). DNA sequence of the construct was compared and confirmed by sequence analysis (Eurofins Genomics GmbH). Recombinant *Bet v 1* protein was expressed as hexahistidine-tagged proteins in *E. coli* BL21 Gold (DE3) (Agilent Technologies). *E. coli* cell pellets containing soluble *rBet v 1* was lysed in 50 mM NaH2PO4, 100 mM NaCl, 10 mM Imidazole, pH 8.0 and lysates were centrifuged for 20 min at 4°C, 10,000 rpm. r*Bet v 1*-containing supernatants were purified by Ni-NTA Agarose affinity chromatography (Qiagen). Protein was eluted in elution buffer with 50 mM NaH2PO4, 100 mM NaCl, 250 mM Imidazole, pH 8.0. Eluted sample was analysed by SDS-PAGE, fractions containing recombinant proteins of more than 90% purity were pooled and dialysed stepwise against 50 mM NaH2PO4, 100 mM NaCl, pH 8.0; 50 mM NaH2PO4, pH 8.0; 40 mM NaH2PO4, pH 8.0; 30 mM NaH2PO4, pH 8.0; and eventually against 20 mM NaH2PO4, pH 8.0. The purified protein was characterised for purity, fold and molecular mass by SDS-PAGE, circular dichroism, mass spectrometry, and ELISA for IgE reactivity.

Statistical analysis

Statistical analysis was performed using R (v4.3.1), R Studio (v2023.06.2+561), and GraphPad Prism (v8.0.0). Normality was assessed with Shapiro-Wilk test (α = 0.05). Non-parametric data were analysed with Mann-Whitney U test or Kruskal-Wallis tests (Dunn’s correction). Two-group comparisons used Mann-Whitney U test or unpaired t-test as appropriate. Spearman’s correlation coefficient (ρ) assessed correlations between RQLQ and antibody concentrations. Degranulation inhibition is expressed as mean ± SD, analysed by one-way ANOVA with Tukey's post hoc test. CAST ELISA performed on leukocytes from birch pollen-allergic patients was analysed by 2-way ANOVA with Dunnett’s multiple comparison test. Serum IgE concentrations (kUA/L) were converted to µg/L by multiplying by 2.4 [13]. Significant differences are annotated with exact P values or asterisks: * p < 0.05; ** p < 0.01; *** p < 0.001; **** p < 0.0001.

Supplementary Tables

Suppl. Table S1. Individual patient characteristics such as AIT cycle, age, ImmunoCAP measurements, RQLQ score, and percentage of RBL degranulation inhibition by patient serum.

| Pat. ID | Bet v 1 allergy | Cohort | AIT | AIT cycle | Age | sIgE [kUa/L] | CAP class | tIgE [kU/L] | sIgG [ug/L] | sIgG4 [ug/L] | RQLQ | RBL in-hib. (%) |
| --- | --- | --- | --- | --- | --- | --- | --- | --- | --- | --- | --- | --- |
| 2 | Yes | Allergic | None | n.a. | 28 | 19.8 | 4 | 186.76 | 2.23 | 0.1866 | 62 | 0 |
| 4 | Yes | Allergic | None | n.a. | 32 | 1.45 | 2 | 61.84 | 1.64 | 0.0404 | 101 | 0 |
| 5 | Yes | Allergic | None | n.a. | 29 | 6.35 | 3 | 36.67 | 1.26 | 0.2649 | - | 25.49 |
| 6 | Yes | Allergic | None | n.a. | 28 | 56.86 | 5 | 386.67 | 1.46 | 0.2069 | 23 | 0 |
| 7 | Yes | Allergic | None | n.a. | 26 | 16.99 | 3 | 31.19 | 1.25 | 0.255 | 47 | 36.31 |
| 8 | Yes | Allergic | None | n.a. | 38 | 1.34 | 2 | 99.18 | 5.93 | 0.5198 | 50 | 0 |
| 9 | Yes | Allergic | None | n.a. | 43 | 20.78 | 4 | 369.81 | 1.32 | 0.3518 | - | 0 |
| 10 | Yes | Allergic | None | n.a. | 31 | 2.02 | 2 | 30.77 | 0.88 | 0.0307 | 142 | 0 |
| 12 | Yes | Allergic | None | n.a. | 46 | 3.56 | 3 | 6.93 | 1.39 | 0.1738 | 87 | 0 |
| 13 | Yes | Allergic | None | n.a. | 29 | 1.99 | 2 | 112.92 | 1.57 | 0.0892 | 39 | 0 |
| 14 | Yes | SCIT | SCIT | 1 | 39 | 13.37 | 3 | 51.9 | 3.41 | 0.403 | 49 | 0 |
| 15 | Yes | SCIT | SCIT | 2 | 42 | 21.92 | 4 | 288.35 | 3.69 | 2.0654 | 0 | 33.87 |
| 16 | Yes | SCIT | SCIT | 2 | 24 | 1.98 | 2 | 26.5 | 0.98 | 0.0565 | 80 | 0 |
| 17 | Yes | SCIT | SCIT | 2 | 21 | 158.79 | 6 | 686.52 | 8.62 | 2.8666 | 33 | 91.63 |
| 18 | Yes | SCIT | SCIT | 2 | 27 | 6.97 | 3 | 15.66 | 3.58 | 0.594 | 25 | 95.41 |
| 20 | Yes | SCIT | SCIT | 2 | 43 | 32.05 | 4 | 528.9 | 1.48 | 0.252 | 60 | 0 |
| 21 | Yes | SCIT | SCIT | 2 | 41 | 5.58 | 3 | 50.71 | 4.95 | 2.8094 | 72 | 98.51 |
| 22 | Yes | SCIT | SCIT | 3 | 36 | 44.11 | 4 | 282.84 | 3.15 | 1.8763 | - | 0 |
| 23 | Yes | SCIT | SCIT | 3 | 36 | 25.3 | 4 | 496.04 | 3.31 | 1.6731 | 10 | 73.54 |
| 24 | Yes | SCIT | SCIT | 3 | 37 | 9.46 | 3 | 39.14 | 2.48 | 1.0801 | 34 | 66.02 |
| 25 | Yes | SCIT | SCIT | 4 | 40 | 31.22 | 4 | 852.54 | 3.72 | 1.8433 | 43 | 73.47 |
| 27 | Yes | SLIT | SLIT | 2 | 49 | 161.22 | 6 | 215.75 | 4.85 | 2.2107 | 92 | 96.04 |
| 28 | Yes | SLIT | SLIT | 2 | 56 | 24.27 | 4 | 132.38 | 5.97 | 2.2369 | 31 | 96.33 |
| 29 | Yes | SLIT | SLIT | 2 | 39 | 11.3 | 3 | 16.31 | 0.81 | 0.0348 | 87 | 26.12 |
| 31 | Yes | SLIT | SLIT | 2 | 34 | 135.35 | 6 | 480.84 | 4.26 | 1.2034 | 7 | 92.44 |
| 32 | Yes | SLIT | SLIT | 2 | 18 | 0.64 | 1 | 94.71 | 0.79 | 0.1026 | - | 0 |
| 34 | Yes | SLIT | SLIT | 2 | 22 | 77.45 | 5 | 190.41 | 4.89 | 0.4867 | - | 87.91 |
| 36 | Yes | SLIT | SLIT | 3 | 47 | 12.74 | 3 | 62.16 | 2.26 | 0.6152 | 10 | 43.7 |
| 37 | Yes | SLIT | SLIT | 3 | 36 | 17.95 | 4 | 201.64 | 3.15 | 1.2628 | 62 | 39.09 |
| 38 | Yes | SLIT | SLIT | 3 | 29 | 0.55 | 1 | 185.65 | 1.05 | 0.0023 | 88 | 0 |
| 39 | No | Non-allergic | n.a. | n.a. | 38 | 0 | 0 | 0.41 | 0.3 | 0 | - | 0 |
| 40 | No | Non-allergic | n.a. | n.a. | 21 | 0.03 | 0 | 320.06 | 0.87 | 0.0105 | 17 | 2 |
| 41 | No | Non-allergic | n.a. | n.a. | 41 | 0 | 0 | 6.74 | 0.7 | 0 | 28 | 0 |
| 43 | No | Non-allergic | n.a. | n.a. | 39 | 0.02 | 0 | 111.09 | 1.1 | 0.0109 | 25 | 0 |
| 44 | No | Non-allergic | n.a. | n.a. | 28 | 0.01 | 0 | 6.67 | 0.56 | 0 | 0 | 0 |

Suppl. Table S2. Different sets of peptides designed for linear and conformational epitope mapping.

| Set # | Mimic type (label) | Description |
| --- | --- | --- |
| 1 | Linear (LIN10) | Linear peptides of length 10 derived from the target sequence of *Bet v 1* protein with an offset of one residue |
| 2 | Linear (LIN15) | Linear peptides of length 15 derived from the target sequence of *Bet v 1* protein with an offset of one residue |
| 3 | Linear (LIN25) | Linear peptides of length 25 derived from the target sequence of *Bet v 1* protein with an offset of one residue |
| 4 | Single loop, mP2 CLIPS (LOOP10) | Constrained peptides of length 10. On positions 2-9 are incorporated 8-mer peptides derived from the target sequence of *Bet v 1* protein with an offset of one residue. Cys residues were inserted on positions 1 and 10 and joined by mP2 CLIPS in order to create a loop mimic. Native Cys are replaced by Cys-acm (denoted “2”) |
| 5 | β-turn peptide mimics, mP2 CLIPS (BET22) | β-turn peptide mimics of length 22. On positions 2-21 are 20-mer peptides derived from the target sequence of *Bet v 1* protein with an offset of one residue. Residues on positions 11 and n12 are replaced by “PG” motif in order to induce the β-turn formation. Cys residues were inserted on positions 1 and 22 and joined by mP2 CLIPS in order to stabilize the mimic. Native Cys are replaced by Cys-acm (denoted “2”). |
| 6 | α-helix, mP2 CLIPS (HEL19) | α-helical peptide mimics of length 19 derived from residues of the target sequence with an offset of one residue. Cys are inserted on positions 1 and 5 and joined by means of mP2 CLIPS to nucleate an α-helical structure. Native Cys are replaced by Cys-acm (denoted “2”). |

Suppl. Table S3. *Bet v 1* sequence used for IgG epitope mapping. We used two versions of the *Bet v 1* allergen. In the second version, the threonine (T) in position 10 was exchanged for an alanine (A). Both isoforms of *Bet v 1* sequences have been reported at the UniProt online platform (P15494): <https://www.uniprot.org/uniprotkb/P15494/entry>.

| **Protein region** | **Amino acid sequence** |
| --- | --- |
| **Bet v 1 (1-60)** | MGVFNYETET TSVIPAARLF KAFILDGDNL FPKVAPQAIS SVENIEGNGG PGTIKKISFP |
| **Bet v 1 (61-120)** | EGFPFKYVKD RVDEVDHTNF KYNYSVIEGG PIGDTLEKIS NEIKIVATPD GGSILKISNK |
| **Bet v 1 (121-160)** | YHTKGDHEVK AEQVKASKEM GETLLRAVES YLLAHSDAYN |

Suppl. Table S4. Main epitope candidates found in this study. Core epitope binders are determined by common sequences in high binding consecutive overlapping peptides. Information is taken from all mimic types, in which the highest intensity mimics are leading.

| Patient sample source | Sample no. | Aa residue no. | Main epitope candidates |
| --- | --- | --- | --- |
| **Allergic (no AIT)** | 2 | *25-34* | DGDNLFPKVA |
|  |  | *58-77* | FPEGFPFKYVKDRVDEVDHT |
|  |  | *98-117* | NEIKIVATPDGGSILKIS |
|  | 6 | *48-55* | GGPGTIKK |
|  |  | *84-91* | SVIEGGPI |
|  |  | *105-119* | VATPDGGSILKISNK |
|  |  | *128-137* | VKAEQVKASK |
|  | 7 | *16-23* | ARLFKAFI |
|  |  | *38-62* | ISSVENIEGNGGPGTIKKISFPEGF |
|  | 8 | *33-42* | VAPQAISSVE |
|  | 13 | *73-80* | EVDHTNFK |
|  |  | *84-91* | SVIEGGPI |
| **Allergic (SCIT)** | 14 | *16-23* | ARLFKAFI |
|  |  | *33-42* | VAPQAISSVE |
|  |  | *89-103* | GPIGDTLEKISNEIK |
|  |  | *91-111* | IGDTLEKISNEIKIVATPDGG |
|  |  | *131-148* | EQVCASKEMGETLLRAVE |
|  | 15 | *74-83* | VDHTNFKYNY |
|  |  | *86-98* | IEGGPIGDTLEKI |
|  |  | *135-141* | ASKEMGE |
|  | 17 | *41-54* | VENIEGNGGPGTIK |
|  |  | *56-66* | ISFPEGFPFKYVK |
|  |  | *87-95* | EGGPIGDTL |
|  |  | *103-116* | KIVATPDGGSILKI |
|  | 18 | *86-93* | IEGGPIGD |
|  | 21 | *85-94* | VIEGGPIGDT |
|  | 23 | *15-24* | ARLFKAFI |
|  |  | *29-43* | LFPKVAPQAISSVEN |
|  |  | *46-55* | GNGGPGTIKK |
|  |  | *86-92* | IEGGPIG |
|  |  | *104-114* | IVATPDGGSIL |
|  |  | *129-138* | KAEQVKASKE |
|  | 24 | *86-98* | IEGGPIGDTLEKI |
|  | 25 | *33-42* | VAPQAISSVE |
|  |  | *72-91* | DEVDHTNFKYNYSVIEGGPI |
| **Allergic (SLIT)** | 27 | *42-48* | ENIEGNG |
|  |  | *70-76* | VDEVDH |
|  |  | *121-132* | HTKGDHEVKAE |
|  | 28 | *62-81* | FPFKYVKDRVDEVDHTNFKY |
|  | 29 | *40-47* | SVENIEGN |
|  |  | *115-122* | KISNKYHT |
|  | 31 | *84-91* | SVIEGGPI |
|  |  | *101-109* | NEIKIVATPD |
|  | 36 | *38-41* | ISSV |
|  |  | *84-91* | SVIEGGPI |
|  |  | *116-125* | ISNKYHTKGD |
|  | 37 | *41-49* | VENIEGNGG |
|  |  | *72-76* | DEVDH |
|  |  | *87-96* | EGGPIGDTLE |
|  |  | *119-128* | KYHTKGDHEV |
|  | 38 | *7-16* | TETTSVIPAA |
|  |  | *84-91* | SVIEGGPI |

**Suppl. Table S5.** Characteristics of *Bet v 1*-derived synthetic peptides. Peptides 1 to 6 with amino acid position and sequence in *Bet v 1*, peptide length of peptide, purity (HLPC) and molecular weight (LC-MS). Putative IgG-binding regions (regions 1 to 4) from epitope mapping are underlined. Peptide 6 are designed using region 3 flanked N-terminally with the aa sequence RV and C-terminally with the aa sequence ISNEIK as to include the IgE-binding epitope.

| **Peptide** | **Position (aa)** | **aa sequence** | **Peptide length** | **Binding region (#)** | **Purity  (%)** | **Molecular weight** |
| --- | --- | --- | --- | --- | --- | --- |
| **1** | 17-46 | Ac-ARLFKAFILD GDNLFPKVAP QAISSVENIE-NH_2_ | 30 | 1 | 98.01 | 3344.87 |
| **2** | 44-73 | Ac-NIEGNGGPGT IKKISFPEGF PFKYVKDRVD-NH_2_ | 30 | 2 | 99.51 | 3351.82 |
| **3** | 74-100 | Ac-EVDHTNFKYN YSVIEGGPIG DTLEK-NH_2_ | 25 | 3 | 99.51 | 2867.12 |
| **4** | 101-130 | Ac-NEIKIVATPD GGSILKISNK YHTKGDHEVK-NH_2_ | 30 | 4 | 99.65 | 3333.80 |
| **5** | 30-59 | Ac-LFPKVAPQAI SSVENIEGNG GPGTIKKISF-NH_2_ | 30 | 1+2 | 95.65 | 3140.64 |
| **6** | 71-105 | Ac-RVDEVDHTNF KYNYSVIEGG PIGDTLEKIS NEIK-NH_2_ | 34 | 3+IgE | 98.20 | 3922.33 |

Supplementary Figures


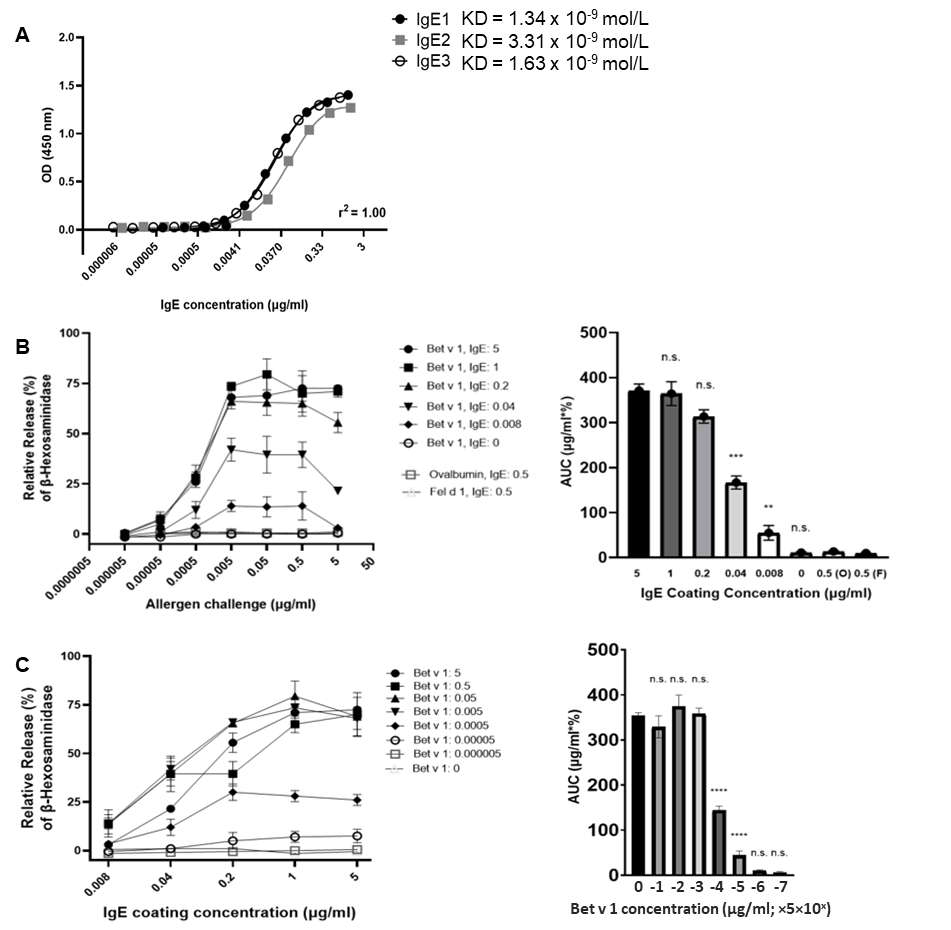


**Suppl. Figure S1.** In vitro model for *Bet v 1* sensitisation and challenge of humanised RBL cells. (**A**) ELISA assay measuring the binding of IgE1, IgE2 and IgE3 to natural *Bet v 1*. The relationship between the concentration of IgE added to wells coated with *Bet v 1* and the measured optical density (OD) at 450 nm, adjusted for the corresponding OD-values measured in non-coated wells, is depicted. The respective KD-value calculated and indicated. (**B**) Mean relative release of β-hexosaminidase from RBL cells sensitised with a fix amount of an equimolar mix of IgE1, IgE2, and IgE3 and challenged with titrated concentrations of natural *Bet v 1* (5×10^−7^ - 5 µg/mL), as well as 0.5 µg/mL of the unrelated allergens ovalbumin or Fel d 1 for specificity control. Each curve represents a different concertation of the IgE mix used for cell sensitisation (0, 0.008, 0.04, 0.2, 1, and 5 μg/mL). The Area Under Curve (AUC) for each test condition was calculated for statistical analysis. (**C**) Relative release of β-hexosaminidase from RBL cells sensitised with titrated concentrations of an equimolar mix of IgE1, IgE2, and IgE3 (0, 0.008, 0.04, 0.2, 1, and 5 μg/mL) and challenged with a fix amount of natural *Bet v 1*. Each curve represents a different concentration of *Bet v 1* (5×10^−7^ - 5 µg/mL). Again, the AUC for each test condition was calculated for statistical analysis. Welch’s ANOVA followed by Dunnett’s T3 Multiple Comparisons Test was performed to test whether each AUC was significantly different from the AUC at the next-highest IgE coating concentration or *Bet v 1* challenge dose.


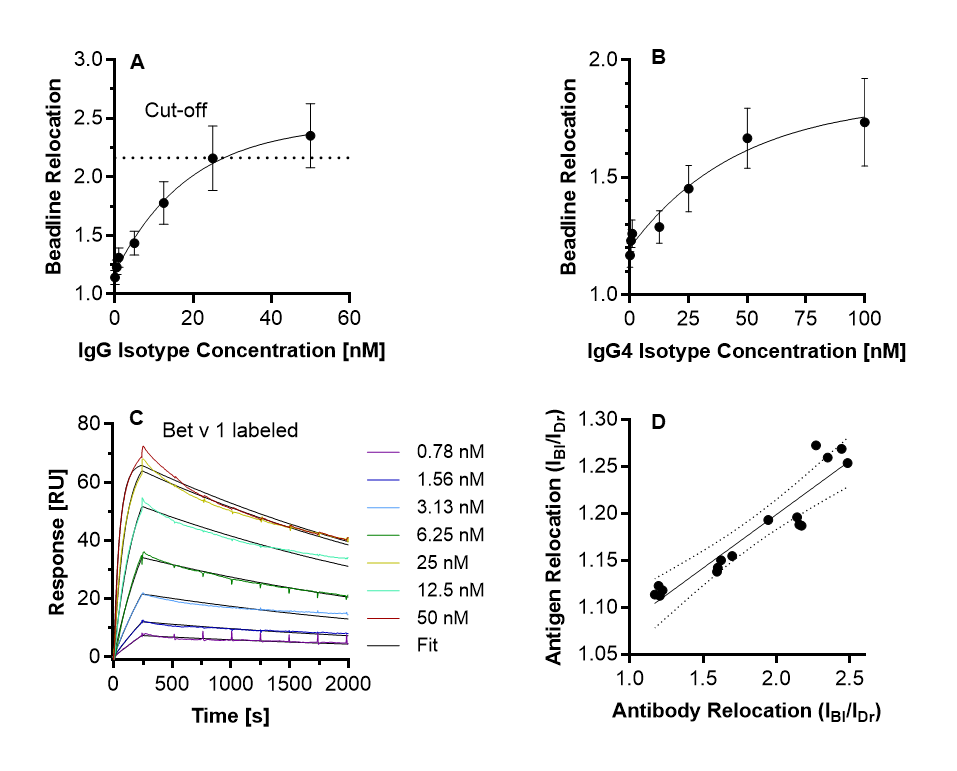


**Suppl. Figure S2. Calbration of DropMap single-cell analysis of the antibody repertoire in allergic patients**. (**A**) Calibration curve for baseline relocation in the Cy5 channel over IgG concentration. Data was fitted using a non-linear one-phase association curve. (**B**) Calibration curve for beadline relocation in the FITC channel over IgG4 concentration. Data was fitted using a non-linear one-phase association curve. (**C**) Antibody affinity of commercial antibody (25 nM) towards *Bet v 1*. (**D**) Antibody binding strength calibration curve with monoclonal mouse anti-*Bet v 1* IgG1 antibody relocation signal on the x-axis and fluorescence-labelled antigen (*Bet v 1*) relocation on the y-axis. A linear fit was applied with a slope of 0.118. The steepness of the slope represents the binding strength of the mAb to *Bet v 1* and was used as a reference for affinity estimation of IgG from patient samples.

**
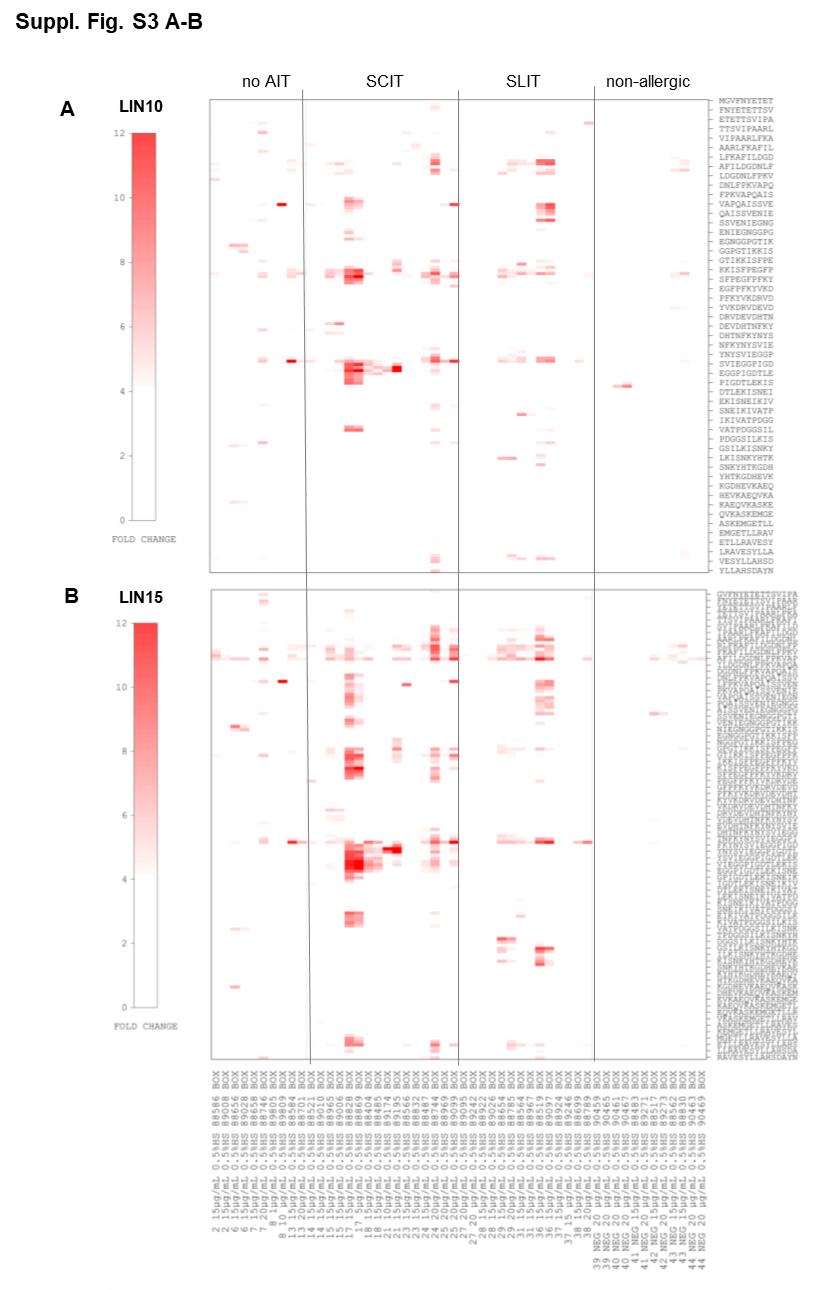
**

**
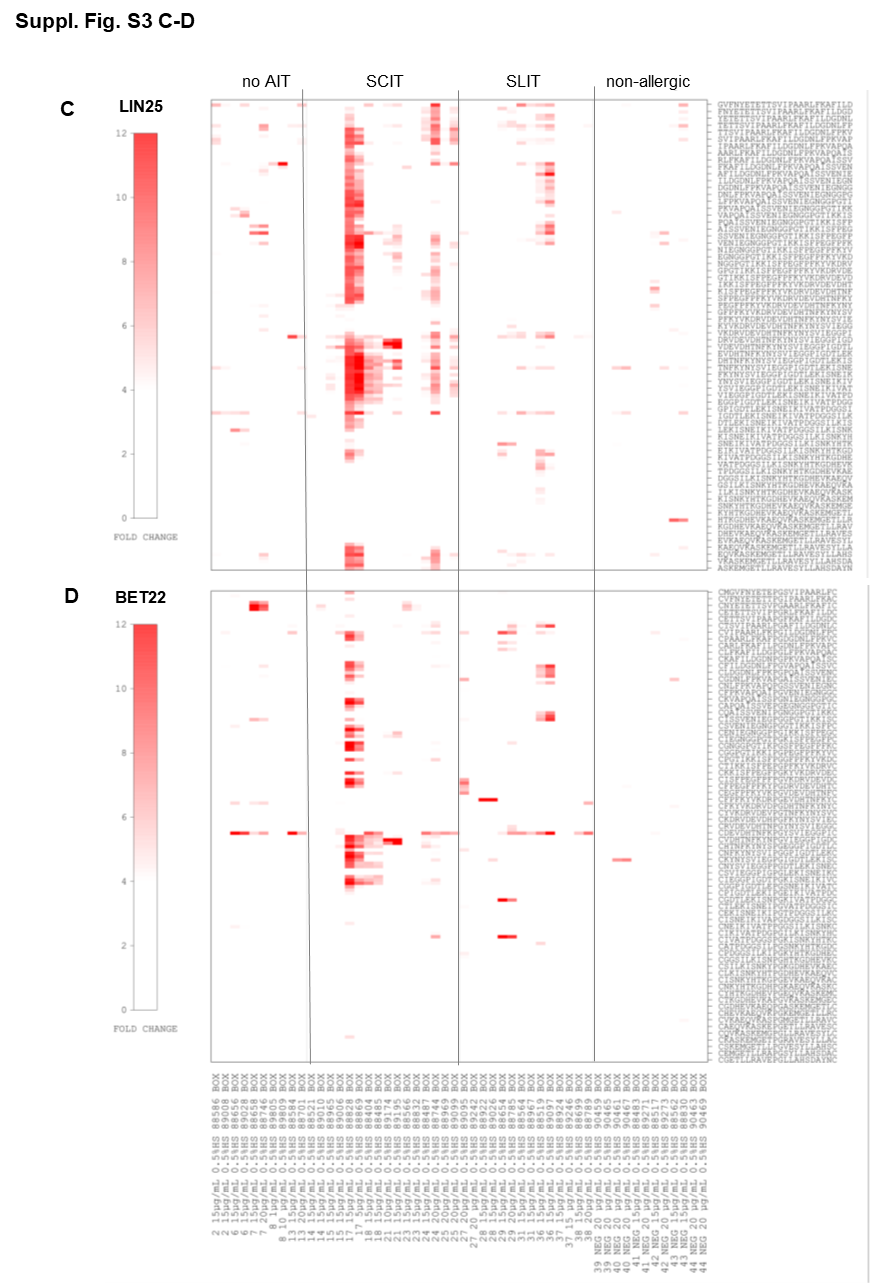
**

**
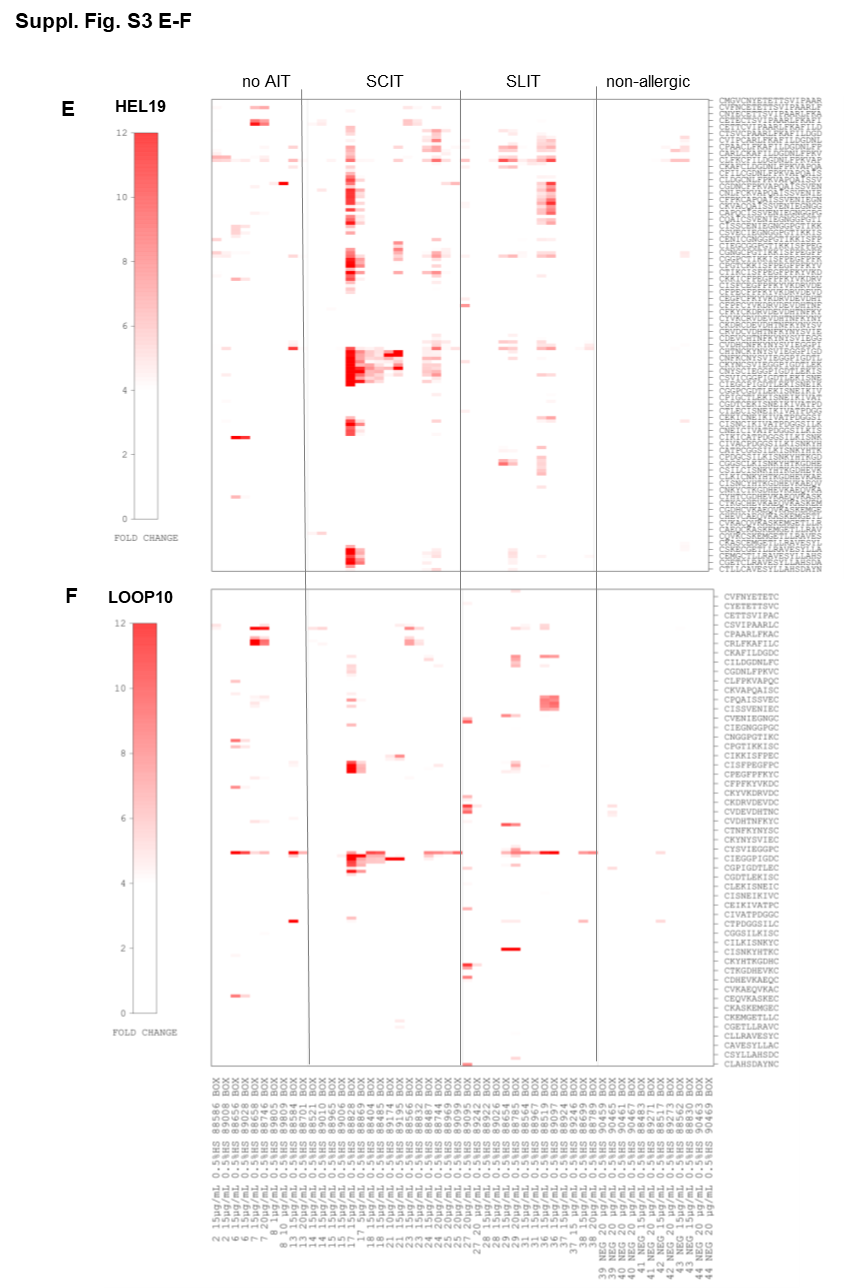
**

**Suppl. Figure S3**. Heatmap analysis of data obtained from all samples on linear and conformational peptides. Samples were screened on linear (**A,B,C**) and conformational (**D,E,F**) peptide arrays of Betv1. Two independent screenings for each sample were plotted for comparison. Peptides exhibiting an intensity at least 4-fold higher than the background (defined as the 10th percentile for the specific peptide mimic) are represented by colours indicated in the FOLD CHANGE colour bar. The sequences of peptides are displayed on the right y-axis. For legibility, only each third peptide sequence is shown. The samples are categorized into the following groups: no-AIT (no allergic immunotherapy), SCIT (subcutaneous immunotherapy), SLIT (sublingual immunotherapy), and non-allergic. LIN, linear; HEL, helical; BET, beta-turn; LOOP, looped.


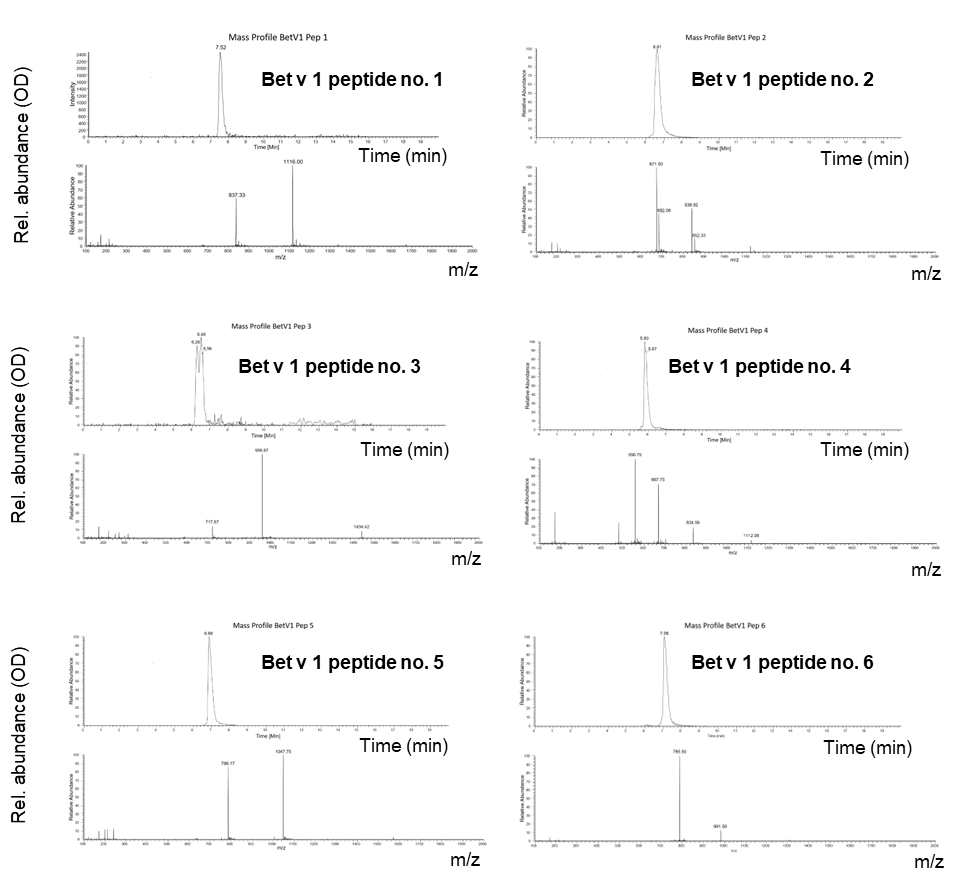


**Suppl. Figure S4. UPLC-MS data and mass profile for all six synthetic peptides.** For each of the six *Bet v 1* peptides, UPLC (*upper panels*) and mass spectrometry (*lower panels*) characteristics determined. The single charged mass cannot be found in the mass spectrum due to the long length of the peptide sequences and thus the high number of positively charged amino acids.


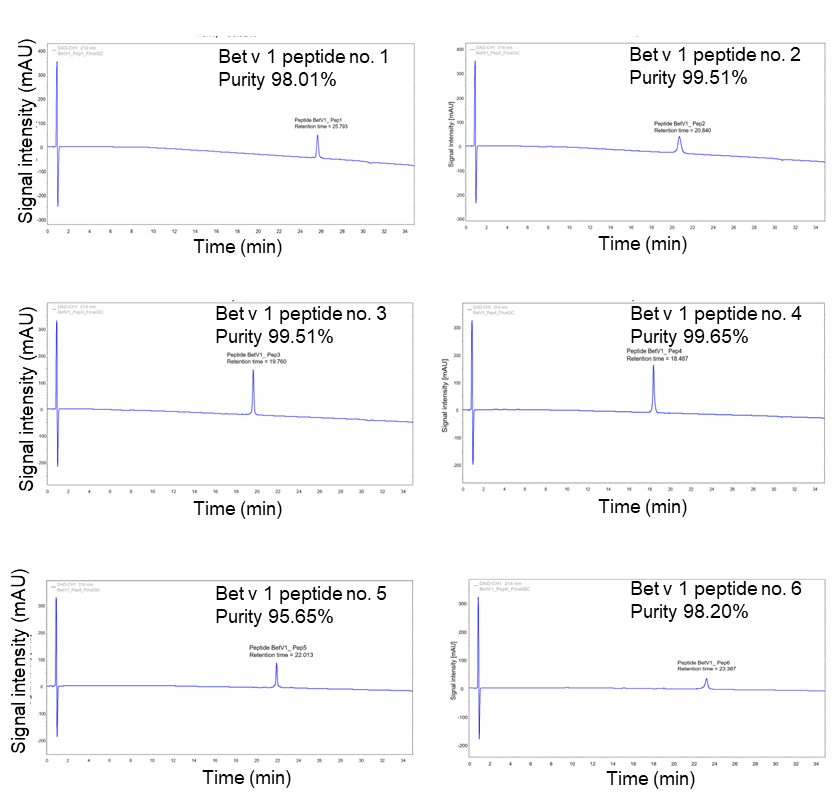


**Suppl. Figure S5.** HPLC-UV spectra of all 6 synthesised peptides. Retention time is indicated in the chromatogram. Purity was calculated as percentage of the total area determined versus the AUC from the peptide. The first peak at the beginning of each chromatogram can be identified as an injection peak and is also found in blanks.

References

1. Juniper EF, Thompson AK, Ferrie PJ, Roberts JN, Validation of the standardized version of the Rhinoconjunctivitis Quality of Life Questionnaire*.* J Allergy Clin Immunol 1999;104: 364-9.

2. Bounab Y, Eyer K, Dixneuf S, Rybczynska M, Chauvel C, Mistretta M, Tran T, Aymerich N, Chenon G, Llitjos JF, Venet F, Monneret G, Gillespie IA, Cortez P, Moucadel V, Pachot A, Troesch A, Leissner P, Textoris J, Bibette J, Guyard C, Baudry J, Griffiths AD, Vedrine C, Dynamic single-cell phenotyping of immune cells using the microfluidic platform DropMap*.* Nat Protoc 2020;15: 2920-55.

3. Aymerich N, Bucheli OTM, Portmann K, Eyer K, Baudry J, A Guide to the Quantitation of Protein Secretion Dynamics at the Single-Cell Level*.* Methods Mol Biol 2024;2804: 141-62.

4. Eyer K, Doineau RCL, Castrillon CE, Briseno-Roa L, Menrath V, Mottet G, England P, Godina A, Brient-Litzler E, Nizak C, Jensen A, Griffiths AD, Bibette J, Bruhns P, Baudry J, Single-cell deep phenotyping of IgG-secreting cells for high-resolution immune monitoring*.* Nat Biotechnol 2017;35: 977-82.

5. Portmann K, Linder A, Oelgarth N, Eyer K, Single-cell deep phenotyping of cytokine release unmasks stimulation-specific biological signatures and distinct secretion dynamics*.* Cell Rep Methods 2023;3: 100502.

6. Timmerman P, Puijk WC, Meloen RH, Functional reconstruction and synthetic mimicry of a conformational epitope using CLIPS technology*.* J Mol Recognit 2007;20: 283-99.

7. Slootstra JW, Puijk WC, Ligtvoet GJ, Langeveld JP, Meloen RH, Structural aspects of antibody-antigen interaction revealed through small random peptide libraries*.* Mol Divers 1996;1: 87-96.

8. Gevaert P, De Craemer J, De Ruyck N, Rottey S, de Hoon J, Hellings PW, Volckaert B, Lesneuck K, Orengo JM, Atanasio A, Kamal MA, Abdallah H, Kamat V, Dingman R, DeVeaux M, Ramesh D, Perlee L, Wang CQ, Weinreich DM, Herman G, Yancopoulos GD, O'Brien MP, Novel antibody cocktail targeting Bet v 1 rapidly and sustainably treats birch allergy symptoms in a phase 1 study*.* J Allergy Clin Immunol 2022;149: 189-99.

9. Orengo JM, Radin AR, Kamat V, Badithe A, Ben LH, Bennett BL, Zhong S, Birchard D, Limnander A, Rafique A, Bautista J, Kostic A, Newell D, Duan X, Franklin MC, Olson W, Huang T, Gandhi NA, Lipsich L, Stahl N, Papadopoulos NJ, Murphy AJ, Yancopoulos GD, Treating cat allergy with monoclonal IgG antibodies that bind allergen and prevent IgE engagement*.* Nat Commun 2018;9: 1421.

10. Atanasio A, Franklin MC, Kamat V, Hernandez AR, Badithe A, Ben LH, Jones J, Bautista J, Yancopoulos GD, Olson W, Murphy AJ, Sleeman MA, Orengo JM, Targeting immunodominant Bet v 1 epitopes with monoclonal antibodies prevents the birch allergic response*.* J Allergy Clin Immunol 2022;149: 200-11.

11. Allenspach MD, Fuchs JA, Doriot N, Hiss JA, Schneider G, Steuer C, Quantification of hydrolyzed peptides and proteins by amino acid fluorescence*.* J Pept Sci 2018;24: e3113.

12. Streuli A, Coxon CR, Steuer C, Simultaneous Quantification of Commonly Used Counter Ions in Peptides and Active Pharmaceutical Ingredients by Mixed Mode Chromatography and Evaporative Light Scattering Detection*.* J Pharm Sci 2021;110: 2997-3003.

13. Hamilton RG, Adkinson NF, Jr., 23. Clinical laboratory assessment of IgE-dependent hypersensitivity*.* J Allergy Clin Immunol 2003;111: S687-701.
